# Assessing Detection of Children With Suicide-Related Emergencies: Evaluation and Development of Computable Phenotyping Approaches

**DOI:** 10.2196/47084

**Published:** 2023-07-21

**Authors:** Juliet Beni Edgcomb, Chi-hong Tseng, Mengtong Pan, Alexandra Klomhaus, Bonnie T Zima

**Affiliations:** 1 Mental Health Informatics and Data Science (MINDS) Hub, Center for Community Health Semel Institute for Neuroscience and Human Behavior University of California Los Angeles Los Angeles, CA United States; 2 Department of Psychiatry David Geffen School of Medicine, University of California Los Angeles Los Angeles, CA United States; 3 Department of Medicine Statistics Core David Geffen School of Medicine, University of California Los Angeles Los Angeles, CA United States

**Keywords:** child mental health, suicide, self-harm, machine learning, phenotyping

## Abstract

**Background:**

Although suicide is a leading cause of death among children, the optimal approach for using health care data sets to detect suicide-related emergencies among children is not known.

**Objective:**

This study aimed to assess the performance of suicide-related International Classification of Diseases, Tenth Revision, Clinical Modification (ICD-10-CM) codes and suicide-related chief complaint in detecting self-injurious thoughts and behaviors (SITB) among children compared with clinician chart review. The study also aimed to examine variations in performance by child sociodemographics and type of self-injury, as well as develop machine learning models trained on codified health record data (features) and clinician chart review (gold standard) and test model detection performance.

**Methods:**

A gold standard classification of suicide-related emergencies was determined through clinician manual review of clinical notes from 600 emergency department visits between 2015 and 2019 by children aged 10 to 17 years. Visits classified with nonfatal suicide attempt or intentional self-harm using the Centers for Disease Control and Prevention surveillance case definition list of ICD-10-CM codes and suicide-related chief complaint were compared with the gold standard classification. Machine learning classifiers (least absolute shrinkage and selection operator–penalized logistic regression and random forest) were then trained and tested using codified health record data (eg, child sociodemographics, medications, disposition, and laboratory testing) and the gold standard classification. The accuracy, sensitivity, and specificity of each detection approach and relative importance of features were examined.

**Results:**

SITB accounted for 47.3% (284/600) of the visits. Suicide-related diagnostic codes missed nearly one-third (82/284, 28.9%) and suicide-related chief complaints missed more than half (153/284, 53.9%) of the children presenting to emergency departments with SITB. Sensitivity was significantly lower for male children than for female children (0.69, 95% CI 0.61-0.77 vs 0.84, 95% CI 0.78-0.90, respectively) and for preteens compared with adolescents (0.66, 95% CI 0.54-0.78 vs 0.86, 95% CI 0.80-0.92, respectively). Specificity was significantly lower for detecting preparatory acts (0.68, 95% CI 0.64-0.72) and attempts (0.67, 95% CI 0.63-0.71) than for detecting ideation (0.79, 95% CI 0.75-0.82). Machine learning–based models significantly improved the sensitivity of detection compared with suicide-related codes and chief complaint alone. Models considering all 84 features performed similarly to models considering only mental health–related ICD-10-CM codes and chief complaints (34 features) and models considering non–ICD-10-CM code indicators and mental health–related chief complaints (53 features).

**Conclusions:**

The capacity to detect children with SITB may be strengthened by applying a machine learning–based approach to codified health record data. To improve integration between clinical research informatics and child mental health care, future research is needed to evaluate the potential benefits of implementing detection approaches at the point of care and identifying precise targets for suicide prevention interventions in children.

## Introduction

### Background

In the United States, suicide is the second leading cause of death among children aged 10 to 14 years, and 1 in 13 children attempts suicide before adulthood [1,2]. Emergency departments are often the first point of access to mental health care for children at risk for suicide, and >1.12 million pediatric emergency department visits each year are suicide related [3-5]. Emergency department visits for self-harm among children tripled between 2007 and 2016 [6], and visits for suicide attempts further increased during the pandemic, particularly among girls and older children [7]. The concurrent rapid growth of health informatics has brought promise that comprehensive clinical data from health records can be used to detect care for suicide-related emergencies in a timely and accurate manner [8-10]. However, the optimal approach to detecting childhood-onset self-injurious thoughts and behaviors (SITB) using health record data remains unknown.

Medical records provide an expanding repository of clinical and phenotypic data to enable low-cost population-based studies on a large scale [11] and inform targeted point-of-care interventions [12]. The discovery of individuals with specific health conditions from within health record data sets historically relied on laborious and time-intensive manual chart review [13]. In recent years, algorithms to classify child psychiatric disorders and adverse childhood experiences have demonstrated the capacity to distinguish cases from noncases using semiautomated approaches to structured codified data (eg, demographics, diagnostic codes, and medications) and text mining with natural language processing [14,15]. Phenotype algorithms currently exist for many childhood-onset mental health conditions, including pediatric depression [16], anxiety [17], developmental language disorder [18], attention-deficit/hyperactivity disorder [15], and autism [19], as well as general pediatric conditions such as Crohn's disease [20], sepsis [21], leukemia and lymphoma [22], and pulmonary hypertension [23].

Nevertheless, little is known about whether the detection of suicide-related emergency department visits using medical record data can be improved through the development and application of phenotype algorithms. Children experience heterogenous manifestations of suicidal thoughts and behaviors across the developmental continuum, and the codified health data elements that differentiate children with SITB from those without are not well characterized. Most surveillance applications exclude children or combine children with adults [24-26]. Trade-offs in current approaches to detecting SITB in children are likely but remain unmeasured. For example, whether suicide-related International Classification of Diseases, Tenth Revision, Clinical Modification (ICD-10-CM), codes and suicide-related chief complaints are sufficiently sensitive and specific in detecting SITB in childhood. Machine learning–based approaches have supported the generation of other clinical phenotypes informative for predicting prognosis, enhancing clinical monitoring, detecting comorbid developmental conditions, and selecting effective treatments [27]. However, the relative benefits of using these approaches are not known for childhood-onset SITB. A recent study distinguishing children with suicidal thoughts and behaviors from those without used data from the Adolescent Brain Cognitive Development study and identified factors difficult to capture using health records: prodromal psychosis, family conflict, depression severity, and impulsivity [28]. Although there is increasing recognition of disparities in predicting suicide events using health records [29], variation in the accuracy of detection of SITB across pediatric population strata (sex, age, race, and ethnicity) remains scarcely described. Knowing which children with SITB are missed by existing approaches could inform efforts to improve detection in an equitable manner and mitigate inequity in the targeted identification of suicide precursors.

### Objectives

To address the aforementioned gaps, the study objectives were to (1) compare the detection performance of suicide-related ICD-10-CM codes and chief complaint with that of clinician manual chart review, (2) examine variations in the detection performance by child sociodemographics and type of SITB (suicidal thoughts, preparatory acts, suicide attempt, and nonsuicidal self-injury), and (3) sequentially train and test a series of phenotype algorithms (machine learning classifiers) to detect SITB using codified health record data of varying complexity.

## Methods

### Design

This was a cross-sectional observational study of emergency department visits by children aged 10 to 17 years. The primary outcome was the classification of the presence or absence of SITB at the emergency department visit. The classification performance of codified medical record data (structured data elements) was compared with that of expert classification by clinician manual chart review of medical records. Algorithmic detection considering three sets of structured data elements was compared with detection considering suicide-related ICD-10-CM codes and suicide-related chief complaint alone (comparator) and chart review (gold standard): (1) mental health–related codes and mental health–related chief complaints, (2) suicide-related codes and non–ICD-10-CM code data elements (ie, other sociodemographic and clinical characteristics of the child), and (3) all structured data elements.

The study followed the STROBE (Strengthening the Reporting of Observational Studies in Epidemiology) statement guidelines.

### Ethics Approval

The study was approved by the University of California Los Angeles institutional review board (20-001512).

### Data Source

The data source was a large university hospital health system comprising 4 hospitals (1 pediatric, 2 medical, and 1 psychiatric) across 2 sites (a tertiary academic medical center and a community hospital). For each child meeting the inclusion criteria, all emergency department medical records were delivered to the study team by the Clinical and Translational Science Institute from the Integrated Clinical and Research Data Repository, a large-scale clinical data warehouse that supports data analyses and extractions for research. The academic medical center site is a primary teaching hospital in Los Angeles, California. This site includes a colocated affiliated children’s hospital and an independently accredited psychiatric hospital with 3 inpatient child psychiatric units serving children with mental illnesses and developmental disabilities. The academic medical center is staffed 24/7 with child and adolescent psychiatrists and general psychiatrists. The community hospital is affiliated with a 25-bed general inpatient pediatric ward. At the community hospital site, children with acute psychiatric complaints are seen by emergency department physicians and licensed clinical social workers.

### Sampling

**Figure 1:**
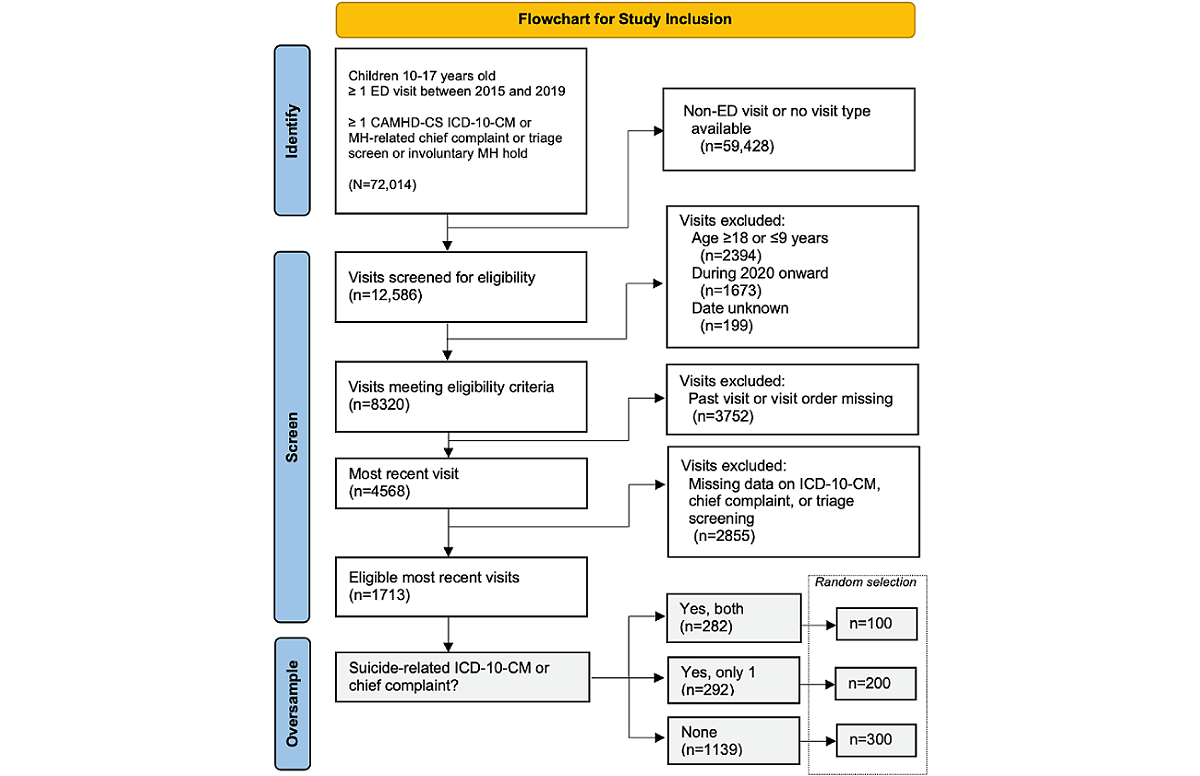
Flowchart of study inclusion. CAMHD-CS: Child and Adolescent Mental Health Disorders Classification System; ED: emergency department; ICD-10-CM: International Classification of Diseases, Tenth Revision, Clinical Modification; MH: mental health.

The flowchart of study inclusion is presented in Figure 1. A series of selection rules were applied to yield a sample feasible for chart review (n=600) and consistent with judicious oversampling informative cases [30]. Visits were restricted to the most recent mental health–related emergency department visit by each child, occurring between October 1, 2015, and October 1, 2019, and defined as emergency department encounters associated with (1) one or more diagnostic code as defined by the Child and Adolescent Mental Health Disorders Classification System (CAMHD-CS) [31]; (2) a mental health–related chief complaint; (3) a positive response to the triage screening question; “Does this patient have a primary psychiatric complaint or suspicion of psychiatric illness?”; or (4) an involuntary mental health detainment order. The final sample was intentionally structured to approximate an equal distribution of 50% cases and 50% noncases. Consequently, from the pool of children who met the inclusion criteria (n=1713), we randomly selected (1) a total of 35.4% (100/282) of children who had both a suicide-related code and a chief complaint, (2) a total of 68.5% (200/292) of children with either a suicide-related code or a chief complaint, and (3) a total of 26.3% (300/1139) of children with neither a suicide-related ICD-10-CM code nor suicide-related a chief complaint. Given the rigorous sampling strategy, a statistical comparison was conducted between the eligible children and those included in the study, and the results are presented in Multimedia Appendix 1. The only significant difference observed was a marginally higher representation of Hispanic or Latinx children in the final sample (27% compared with 24% in the sample of eligible children; *P*=.02).

### Study Variable Construction

Sociodemographics included child age, natal sex, race, and ethnicity. These variables were self- or parent-reported at the point of care. Socioeconomic disadvantage was assessed using the Area Deprivation Index (ADI) [32]. The Federal Information Processing System (FIPS) code of each child’s home address was linked to the ADI with decile ranked at the state and national levels. The only variable for which missing values were present was ADI (missing for 56/600, 9% children), and missing values for ADI were imputed through corresponding medians. Additional structured data indicators were considered (eg, gender identity, family history, and language) but omitted owing to sparsity.

Clinical characteristics included diagnostic or billing codes, chief complaint, orders (medications, laboratory tests, and involuntary hold status), site (academic medical center vs community hospital), and prior care use. All mental health–related diagnostic or billing codes (ICD-10-CM) from the emergency department visit were categorized using the CAMHD-CS [31]. The presence of an ICD-10-CM code for SITB was determined by the presence of one or more codes from the Centers for Disease Control and Prevention (CDC) surveillance case definition list [24] and associated with the emergency department visit. Of note, the codes used to assign the CAMHD-CS category of suicide or self-injury align exactly with the CDC code list. The chief complaint for SITB was determined by the selection of *suicidal* or *suicide attempt* by nursing triage upon the child’s arrival at the emergency department. Laboratory tests were restricted to those ordered and collected during the emergency department visit and included those related to overdose (serum acetaminophen, salicylates, benzodiazepines, and tricyclics), urine drug screen results, and serum alcohol. All psychotropic medications (n=97) received during the visit were consolidated using the Anatomical Therapeutic Chemical classification system into 8 categories (antidepressants, antiepileptics, antihistamines, antipsychotics, anxiolytics, hypnotics and sedatives, lithium, and psychostimulants). Additional clinical characteristics were encounter year, site, emergency department disposition, provider sex, as well as the child’s number of prior 90-, 180-, and 365-day emergency department visits and general medical and psychiatric hospitalizations. A full list of sociodemographic and clinical characteristics and definitions are included in Multimedia Appendix 2.

### Manual Chart Abstraction

All clinical notes from each emergency department visit were extracted and provided to the study team verbatim. The notes included physician history and physical examinations, progress notes, social work notes, and nursing notes.

Classification by the manual review of records was adapted from the Columbia Classification Algorithm of Suicide Assessment (C-CASA) [33]. The C-CASA is a system for categorizing suicide-related behavior that takes into account research-based definitions of suicidality and has been applied to the classification of emergency presentations for children [34]. The criteria for defining a suicide attempt include both self-harm and intent to die [33]. Including intent in the definition of suicide helps distinguish between those who engage in self-harm with the intent to die and those who do so for other reasons. The C-CASA has 8 categories that differentiate among suicidal behavior, nonsuicidal behavior, and behavior that is potentially suicidal.

Consistent with operationalized guidelines for C-CASA, if >1 category was present, the abstractor coded the visit as consistent with the most severe category: suicide death, nonfatal attempt, preparatory behavior, suicidal ideation, self-injurious behavior intent unknown, not enough information, and self-injurious behavior without suicidal intent [33]. To capture cases with combined nonsuicidal self-injurious behavior and suicidal thoughts or behaviors, the classification system was adapted to specify the presence and type of self-injurious behavior (intent unknown or no suicidal intent) in a secondary classification field.

Classifications were compared and differed in only 0.3% (2/600) of the cases for presence or absence of SITB. Classifications differed in 2.5% (15/600) of the cases for type of SITB. In the second stage, a second board-certified child psychiatrist (BZ) and child psychiatric nurse practitioner (KC), also blinded, separately reviewed all discordant cases. Of the discordant cases for which concordance was not reached (4/600, 0.6%), consensus discussion yielded a final classification.

### Analyses

#### Rule-Based Classification

Contingency matrixes were constructed comparing classification with suicide-related ICD-10-CM code and suicide-related chief complaint (comparator) and manual chart review (gold standard). The sensitivity, specificity, and accuracy were calculated, with 95% CIs computed using Clopper-Pearson CIs. Variations in performance by demographics were examined by subsetting the sample by demographic characteristics (eg, male children). Variations in detection performance for type of SITB (eg, suicidal ideation) were examined by comparing classification using structured data elements with classification of type upon manual chart review.

#### Machine Learning–Based Classification

Fit metrics were measured via 10-fold cross-validation. For each fold, a machine learning model was trained with structured data elements (features) and the manual chart review (gold standard) for each child in a training set. Next, this model was used to classify the presence or absence of SITB (predicted outcome) of each child in a test set, and this predicted outcome was compared with the manual chart review (gold standard) to yield fit metrics. CIs for fit metrics were calculated by examining the variations in fit metrics across the test sets.

Three sets of structured data elements were compared, representing varying levels of complexity of codified health record data: (1) mental health–related ICD-10-CM codes and mental health–related chief complaints (34 features); (2) suicide-related ICD-10-CM codes and all child sociodemographics and clinical characteristics, excluding mental health–related ICD-10-CM codes (53 features); and (3) all structured data elements (84 features). The first set was chosen to evaluate classification performance using mental health–related ICD-10-CM codes and chief complaints to detect cases. The second set was used to determine the relative importance of considering other, non–ICD-10-CM–based structured data elements (ie, how well detection can be performed without mental health comorbidity codes). Variables in the first and second sets are mutually exclusive, except for suicide-related ICD-10-CM codes and chief complaints, which are included in both sets. The third set was used to comprehensively evaluate the structured data elements that might support the detection of cases and to test the optimization of detection using a broad set of codified data.

Two classifier types were compared: least absolute shrinkage and selection operator (LASSO)–penalized logistic regression (hereinafter referred to as LASSO) and random forest. LASSO was selected to perform variable selection and yield a parsimonious model involving only a subset of variables relevant to the classification task [35]. Random forest was selected to stratify the predictor space and produce a consensus prediction using an ensemble of decision trees [36]. LASSO and random forest were selected because both are well documented in the informatics literature and widely used for phenotyping applications [37]. Fit metrics were compared using McNemar chi-square tests. The classifiers were anticipated to have predictive ability, with accuracy ranging from 70% to 95%. Given the study sample size, the margin of error was estimated to be <4%.

Feature engineering was conducted using R statistical software (version 4.2.0; R Foundation for Statistical Computing), and the models were implemented using Python (version 3.12; Python Software Foundation) with *scikit-learn* (version 1.2.2) toolboxes *sklearn.linear_model.lasso*, *sklearn.ensemble.RandomForestClassifier*, and *sklearn.metrics*. Hyperparameters were set to default and were as follows: LASSO-penalized logistic regression (L1 penalty, *liblinear* solver, and regularization score 1.0) and random forest (100 trees, bootstrap samples, Gini impurity for tree split quality, and no balancing or class weights). The random forest was run with out-of-bag samples to estimate generalization error. A set seed was used to ensure replicability. The code is available from the authors upon request.

#### Sampling Probability Adjustment

As the study population was a stratified random subsample of the total population, we compared rule-based classification fit metrics, both with and without the adjustment for sampling probability. The adjustment was performed by considering the subsample as a stratified 2-phase sample and applying inverse probability weighting. Further detail on this method is described by Katki et al [30].

## Results

### Sample Characteristics

Child sociodemographics and clinical characteristics are presented in Table 1. Additional sample characteristics are described in Multimedia Appendices 3 and 4.

**Table 1:** Sample characteristics (n=600).

				Values, n (%)
**Sex**				
	Male			276 (46)
	Female			324 (54)
**Age group (years)**				
	10-12.9			115 (19.2)
	13-15.9			215 (35.8)
	16-17.9			270 (45)
**Race**				
	American Indian or Alaska Native			2 (0.3)
	Asian			35 (5.8)
	Black or African American			61 (10.2)
	Native Hawaiian or other Pacific Islander			0 (0)
	White			323 (53.8)
	Other^a^			127 (21.1)
**Ethnicity**				
	Hispanic or Latinx			161 (26.8)
	Not Hispanic or Latinx			390 (65)
	Other^a^			3 (0.5)
**State ADI^b^ decile**				
	1-3			333 (55.5)
	4-6			119 (19.8)
	7-10			92 (15.3)
	Missing			56 (9.3)
**Site**				
	Academic medical center			455 (75.8)
	Community hospital			145 (24.2)
**Disposition**				
	Discharged without hospitalization^c^			322 (53.7)
	General medical hospitalization			106 (17.7)
	**Psychiatric hospitalization**			
		Within health system	134 (22.3)	
		Transferred outside health system	38 (6.3)	
**Legal status**				
	72-hour hold (involuntary)			123 (20.5)
	Voluntary			477 (79.5)
**Chief complaint**				
	Psychiatric (including suicide related)			370 (61.7)
	Suicide related			131 (21.8)
	Other			227 (37.8)
**Top 10 diagnostic code groups^d^**				
	Depressive disorders			221 (36.8)
	Suicide or self-injury			203 (33.8)
	Anxiety disorders			181 (30.2)
	Attention deficit hyperactivity disorder			105 (17.5)
	Substance-related and addictive disorders			80 (13.3)
	Mental health symptom			76 (12.7)
	Autism spectrum disorder			59 (9.8)
	Disruptive, impulse control, and conduct disorders			35 (5.8)
	Obsessive-compulsive and related disorders			33 (5.5)
	Trauma and stressor-related disorders			32 (5.3)
	Bipolar and related disorders			24 (4)

^a^Multiple races, not available, other, patient refused, or unknown.

^b^ADI: Area Deprivation Index.

^c^Eloped (4/322, 1.2%), left without being seen (2/322, 0.6%), left against medical advice (2/322, 0.6%), inpatient rehabilitation facility (3/422, 0.9%), law enforcement (1/322, 0.3%), skilled nursing (1/322, 0.3%), and expired (3/322, 0.9%).

^d^Ten most prevalent Child and Adolescent Mental Health Disorders Classification System (CAMHD-CS) diagnostic code groups, in order of prevalence in study sample.

### Performance of Rule-Based Classification

The detection performance of suicide-related ICD-10-CM codes and chief complaints compared with that of manual chart review is presented in Table 2. Manual chart review labeled 47.3% (284/600) of the visits as consistent with SITB (gold standard positive). Classification using suicide-related codes alone resulted in 85 false negatives with sensitivity 0.70, specificity 0.99, and accuracy 0.85. Classification using suicide-related chief complaint alone resulted in 155 false negatives with sensitivity 0.45, specificity 0.99, and accuracy 0.74. The highest misclassification was observed if a suicide-related code and a suicide-related chief complaint were necessary to classify the visit as SITB positive (sensitivity 0.38, specificity 1.00, and accuracy 0.71). The lowest misclassification rate was observed if either a suicide-related code or a suicide-related chief complaint classified the visit as SITB positive (sensitivity 0.77, specificity 0.98, and accuracy 0.89). The sensitivity of suicide-related codes and suicide-related chief complaints (either affirmed) was significantly lower among male children (0.69, 95% CI 0.61-0.77) than among female children (0.84, 95% CI 0.78-0.90). Sensitivity was also significantly lower in detecting cases of SITB among those aged 10 to 12 years (0.66, 95% CI 0.54-0.78) than among those aged 13 to 15 years (0.86, 95% CI 0.80-0.92). Differences in fit metrics by race and ethnicity did not reach statistical significance. There were no substantial differences between adjusted and unadjusted fit metrics, and sampling probability–adjusted estimates are included in Multimedia Appendix 5.

Detection performance by type of SITB is presented in Table 3. The sensitivity of detection did not differ by type. For suicide-related codes and suicide-related chief complaints (either affirmed), the specificity of detection was significantly lower for preparatory acts (0.68, 95% CI 0.64-0.72) and suicide attempts (0.67, 95% CI 0.63-0.71) than for suicidal ideation (0.79, 95% CI 0.75-0.82). There were no substantial differences between adjusted and unadjusted fit metrics, and sampling probability–adjusted estimates are included in Multimedia Appendix 6.

**Table 2:** Performance of International Classification of Diseases, Tenth Revision, Clinical Modification (ICD-10-CM), code (as defined by the Centers for Disease Control and Prevention case surveillance definition list) and suicide-related chief complaint in detecting cases of self-injurious thoughts and behaviors compared with that of manual chart abstraction: total sample and stratified by natal sex, age group, race, and ethnicity.

Sample and classification				True positive, n (%)		False positive, n (%)		False negative, n (%)		True negative, n (%)		Sensitivity (95% CI)		Specificity (95% CI)		Accuracy (95% CI)
**All (n=600)**																
	ICD-10-CM			199 (33.2)		4 (0.7)		85 (14.2)		312 (52)		0.70 (0.65-0.75)		0.99 (0.98-1.00)		0.85 (0.82-0.88)
	CC^a,b^			129 (21.5)		2 (0.3)		155 (25.8)		314 (52.3)		0.45 (0.40-0.51)		0.99 (0.98-1.00)		0.74 (0.70-0.77)
	ICD-10-CM or CC^c^			220 (36.7)		5 (0.8)		64 (10.7)		311 (51.8)		0.77 (0.73-0.82)		0.98 (0.97-1.00)		0.89 (0.86-0.91)
	ICD-10-CM and CC^d^			108 (18)		1 (0.2)		176 (29.3)		315 (52.5)		0.38 (0.32-0.44)		1.00 (0.99-1.00)		0.71 (0.67-0.74)
**Sex**																
	**Male (n=276)**															
		ICD-10-CM	78 (28.3)		0 (0)		45 (16.3)		153 (55.4)		0.63 (0.55-0.72)		1.00 (1.00-1.00)		0.84 (0.79-0.88)	
		CC	52 (18.8)		0 (0)		71 (25.7)		153 (55.4)		0.42 (0.34-0.51)		1.00 (1.00-1.00)		0.74 (0.69-0.79)	
		ICD-10-CM or CC	85 (30.8)		0 (0)		38 (13.8)		153 (55.4)		0.69 (0.61-0.77)		1.00 (1.00-1.00)		0.86 (0.82-0.90)	
		ICD-10-CM and CC	45 (16.3)		0 (0)		78 (28.3)		153 (55.4)		0.37 (0.28-0.45)		1.00 (1.00-1.00)		0.72 (0.66-0.77)	
	**Female (n=324)**															
		ICD-10-CM	121 (37.3)		4 (1.2)		40 (12.3)		159 (49.1)		0.75 (0.68-0.82)		0.98 (0.95-1.00)		0.86 (0.83-0.90)	
		CC	77 (23.8)		2 (0.6)		84 (25.9)		161 (49.7)		0.48 (0.40-0.56)		0.99 (0.97-1.00)		0.73 (0.69-0.78)	
		ICD-10-CM or CC	135 (41.7)		5 (1.5)		26 (8)		158 (48.8)		0.84 (0.78-0.90)		0.97 (0.94-1.00)		0.90 (0.87-0.94)	
		ICD-10-CM and CC	63 (19.4)		1 (0.3)		98 (30.2)		162 (50)		0.39 (0.32-0.47)		0.99 (0.98-1.00)		0.69 (0.64-0.74)	
**Age group (years)**																
	**10-12.9 (n=115)**															
		ICD-10-CM	33 (28.7)		0 (0)		26 (22.6)		56 (48.7)		0.56 (0.43-0.69)		1.00 (1.00-1.00)		0.77 (0.70-0.85)	
		CC	25 (21.7)		0 (0)		34 (29.6)		56 (48.7)		0.42 (0.30-0.55)		1.00 (1.00-1.00)		0.70 (0.62-0.79)	
		ICD-10-CM or CC	39 (33.9)		0 (0)		20 (17.4)		56 (48.7)		0.66 (0.54-0.78)		1.00 (1.00-1.00)		0.83 (0.76-0.90)	
		ICD-10-CM and CC	19 (16.5)		0 (0)		40 (34.8)		56 (48.7)		0.32 (0.20-0.44)		1.00 (1.00-1.00)		0.65 (0.57-0.74)	
	**13-15.9 (n=215)**															
		ICD-10-CM	90 (41.9)		1 (0.5)		26 (12.1)		98 (45.6)		0.78 (0.70-0.85)		0.99 (0.97-1.00)		0.87 (0.83-0.92)	
		CC	57 (26.5)		1 (0.5)		59 (27.4)		98 (45.6)		0.49 (0.40-0.58)		0.99 (0.97-1.00)		0.72 (0.66-0.78)	
		ICD-10-CM or CC	100 (46.5)		2 (0.9)		16 (7.4)		97 (45.1)		0.86 (0.80-0.92)		0.98 (0.95-1.00)		0.92 (0.88-0.95)	
		ICD-10-CM and CC	47 (21.9)		0 (0)		69 (32.1)		99 (46)		0.41 (0.32-0.49)		1.00 (1.00-1.00)		0.68 (0.62-0.74)	
	**16-17.9 (n=270)**															
		ICD-10-CM	76 (28.1)		3 (1.11)		33 (1.5)		158 (58.5)		0.70 (0.61-0.78)		0.98 (0.96-1.00)		0.87 (0.83-0.91)	
		CC	47 (17.4)		1 (0.4)		62 (23)		160 (59.3)		0.43 (0.34-0.52)		0.99 (0.98-1.00)		0.77 (0.72-0.82)	
		ICD-10-CM or CC	81 (30)		3 (1.11)		28 (10.4)		158 (58.5)		0.74 (0.66-0.83)		0.98 (0.96-1.00)		0.89 (0.85-0.92)	
		ICD and CC	42 (15.6)		1 (0.4)		67 (24.8)		160 (59.3)		0.39 (0.29-0.48)		0.99 (0.98-1.00)		0.75 (0.70-0.80)	
**Race and ethnicity**																
	**Asian, non-Hispanic (n=35)**															
		ICD-10-CM	12 (34.3)		0 (0)		3 (8.6)		20 (57.1)		0.8 (0.6-1.00)		1.00 (1.00-1.00)		0.82 (0.91-1.00)	
		CC	5 (14.3)		0 (0)		10 (28.6)		20 (57.1)		0.33 (0.09-0.57)		1.00 (1.00-1.00)		0.56 (0.71-0.86)	
		ICD-10-CM or CC	13 (37.1)		0 (0)		2 (5.7)		20 (57.1)		0.87 (0.69-1.00)		1.00 (1.00-1.00)		0.87 (0.94-1.00)	
		ICD-10-CM and CC	4 (11.4)		0 (0)		11 (31.4)		20 (57.1)		0.27 (0.04-0.49)		1.00 (1.00-1.00)		0.69 (0.53-0.84)	
	**Black, non-Hispanic (n=61)**															
		ICD-10-CM	21 (34.4)		0 (0)		10 (16.4)		30 (49.2)		0.68 (0.51-0.84)		1.00 (1.00-1.00)		0.84 (0.74-0.93)	
		CC	12 (19.7)		0 (0)		19 (31.1)		30 (49.2)		0.39 (0.22-0.56)		1.00 (1.00-1.00)		0.57 (0.69-0.80)	
		ICD-10-CM or CC	23 (37.7)		0 (0)		8 (13.1)		30 (49.2)		0.74 (0.59-0.90)		1.00 (1.00-1.00)		0.78 (0.87-0.95)	
		ICD-10-CM and CC	10 (16.4)		0 (0)		21 (34.4)		30 (49.2)		0.32 (0.16-0.49)		1.00 (1.00-1.00)		0.66 (0.54-0.77)	
	**Hispanic or Latinx (n=161)**															
		ICD-10-CM	44 (27.3)		1 (0.6)		25 (15.5)		91 (56.5)		0.64 (0.52-0.75)		0.99 (0.97-1.00)		0.84 (0.78-0.90)	
		CC	32 (19.9)		1 (0.6)		37 (23)		91 (56.5)		0.46 (0.35-0.58)		0.99 (0.97-1.00)		0.76 (0.70-0.83)	
		ICD-10-CM or CC	51 (31.7)		2 (1.2)		18 (11.2)		90 (55.9)		0.74 (0.64-0.84)		0.98 (0.95-1.00)		0.82 (0.88-0.93)	
		ICD-10-CM and CC	25 (15.5)		0 (0)		44 (27.3)		92 (57.1)		0.36 (0.25-0.48)		1.00 (1.00-1.00)		0.66 (0.73-0.80)	
	**White, non-Hispanic (n=285)**															
		ICD-10-CM	106 (37.2)		2 (0.7)		42 (14.7)		135 (47.4)		0.72 (0.64-0.79)		0.99 (0.97-1.00)		0.85 (0.80-0.89)	
		CC	68 (23.9)		1 (0.4)		80 (28.1)		136 (47.7)		0.46 (0.38-0.54)		0.99 (0.98-1.00)		0.72 (0.66-0.77)	
		ICD-10-CM or CC	116 (40.7)		2 (0.7)		32 (11.2)		135 (47.4)		0.78 (0.72-0.85)		0.99 (0.97-1.00)		0.88 (0.84-0.92)	
		ICD-10-CM and CC	58 (20.4)		1 (0.4)		90 (31.6)		136 (47.7)		0.39 (0.31-0.47)		0.99 (0.98-1.00)		0.68 (0.63-0.73)	
	**Other^e^ (n=58)**															
		ICD-10-CM	16 (27.6)		1 (1.7)		5 (8.6)		36 (62.1)		0.76 (0.58-0.94)		0.97 (0.92-1.03)		0.82 (0.90-0.97)	
		CC	12 (20.7)		0 (0)		9 (15.5)		37 (63.8)		0.57 (0.36-0.78)		1.00 (1.00-1.00)		0.75 (0.84-0.94)	
		ICD-10-CM or CC	17 (29.3)		1 (1.7)		4 (6.9)		36 (62.1)		0.81 (0.64-0.98)		0.97 (0.92-1.03)		0.84 (0.91-0.99)	
		ICD-10-CM and CC	11 (19)		0 (0)		10 (17.2)		37 (63.8)		0.52 (0.31-0.74)		1.00 (1.00-1.00)		0.73 (0.83-0.92)	

^a^CC: chief complaint.

^b^CC refers to suicide-related chief complaints.

^c^Cases classified as self-injurious thoughts and behaviors if either a suicide-related ICD-10-CM code or a suicide-related CC was present (either affirmed).

^d^Cases classified as self-injurious thoughts and behaviors if both a suicide-related ICD-10-CM code and a suicide-related CC were present (both affirmed).

^e^American Indian or Alaska Native, Native Hawaiian or Pacific Islander, multiple races, not available, other, patient refused, and unknown.

**Table 3:** Performance of International Classification of Diseases, Tenth Revision, Clinical Modification (ICD-10-CM), code (as defined by the Centers for Disease Control and Prevention case surveillance definition list) and suicide-related chief complaint in detecting cases of self-injurious thoughts and behaviors compared with manual chart abstraction: stratified by Columbia Classification Algorithm of Suicide Assessment categorization (n=600).

Categorization and classification			True positive, n (%)	False positive, n (%)	False negative, n (%)			True negative, n (%)		Sensitivity (95% CI)		Specificity (95% CI)		Accuracy (95% CI)
**Suicidal ideation**														
	ICD-10-CM: broad^a^	105 (17.5)		98 (16.3)		39 (6.5)	358 (59.7)		0.73 (0.66-0.80)		0.79 (0.75-0.82)		0.77 (0.74-0.81)	
	ICD-10-CM: strict^b^	104 (17.3)		87 (14.5)		40 (6.7)	369 (61.5)		0.72 (0.65-0.80)		0.81 (0.77-0.85)		0.76 (0.79-0.82)	
	CC^c,d^	73 (12.2)		58 (9.7)		71 (11.8)	398 (66.3)		0.51 (0.43-0.59)		0.87 (0.84-0.90)		0.75 (0.79-0.82)	
	ICD-10-CM or CC^e^	118 (19.7)		97 (16.2)		26 (4.3)	359 (59.8)		0.82 (0.76-0.88)		0.79 (0.75-0.82)		0.80 (0.76-0.83)	
	ICD-10-CM and CC^f^	60 (10)		49 (8.2)		84 (14)	407 (67.8)		0.42 (0.34-0.50)		0.89 (0.86-0.92)		0.78 (0.75-0.81)	
**Preparatory acts**														
	ICD-10-CM	35 (5.8)		168 (28)		10 (1.7)	387 (64.5)		0.78 (0.66-0.90)		0.70 (0.66-0.74)		0.70 (0.67-0.74)	
	CC	23 (3.8)		108 (18)		22 (3.7)	447 (74.5)		0.51 (0.37-0.66)		0.77 (0.81-0.84)		0.78 (0.75-0.82)	
	ICD-10-CM or CC	36 (6)		179 (29.8)		9 (1.5)	376 (62.7)		0.80 (0.68-0.92)		0.64 (0.68-0.72)		0.69 (0.65-0.72)	
	ICD-10-CM and CC	20 (3.3)		89 (14.8)		33 (5.5)	458 (76.3)		0.38 (0.25-0.51)		0.81 (0.87-0.84)		0.80 (0.76-0.83)	
**Suicide attempt**														
	ICD-10-CM	42 (7)		161 (26.8)		11 (1.8)	386 (64.3)		0.79 (0.68-0.90)		0.71 (0.67-0.74)		0.71 (0.68-0.75)	
	CC	22 (3.7)		109 (18.2)		31 (5.2)	438 (73)		0.42 (0.28-0.55)		0.80 (0.77-0.83)		0.77 (0.73-0.80)	
	ICD-10-CM or CC	44 (7.3)		181 (30.2)		9 (1.5)	366 (61)		0.83 (0.73-0.93)		0.67 (0.63-0.71)		0.67 (0.63-0.71)	
	ICD-10-CM and CC	20 (3.3)		89 (14.8)		33 (5.5)	458 (76.3)		0.38 (0.25-0.51)		0.84 (0.81-0.87)		0.80 (0.77-0.83)	
**Nonsuicidal self-injurious behavior**														
	ICD-10-CM	74 (12.3)		129 (21.5)		35 (5.8)	362 (60.3)		0.68 (0.59-0.77)		0.74 (0.70-0.78)		0.73 (0.69-0.76)	
	CC	47 (7.8)		84 (14)		62 (10.3)	407 (67.8)		0.43 (0.34-0.52)		0.83 (0.80-0.86)		0.76 (0.72-0.79)	
	ICD-10-CM or CC	81 (13.5)		134 (22.3)		28 (4.7)	357 (59.5)		0.74 (0.66-0.83)		0.73 (0.69-0.77)		0.69 (0.73-0.77)	
	ICD-10-CM and CC	37 (6.2)		72 (12)		71 (11.8)	420 (70)		0.34 (0.25-0.43)		0.82 (0.85-0.88)		0.73 (0.76-0.80)	

^a^The entire Centers for Disease Control and Prevention case surveillance definition International Classification of Diseases, Tenth Revision, Clinical Modification, code list was used.

^b^Only the ICD-10-CM code for suicidal ideation (R45.81) was used.

^c^CC: chief complaint.

^d^CC refers to suicide-related chief complaints

^d^Cases classified as self-injurious thoughts and behaviors if either a suicide-related ICD-10-CM code or a suicide-related CC was present (either affirmed).

^e^Cases classified as self-injurious thoughts and behaviors if both a suicide-related ICD-10-CM code and a suicide-related CC were present (both affirmed).

### Performance of Machine Learning–Based Classification

Fit metrics by classifier type and considered features are presented in Table 4. The LASSO and random forest classifiers performed similarly. Classification using only suicide-related codes and suicide-related chief complaints was less sensitive (0.77) and more specific (0.98) than classification using machine learning–based classification (sensitivity 0.84-0.86 and specificity 0.91-0.95). McNemar chi-square tests are presented in Multimedia Appendix 7.

**Figure 2:**
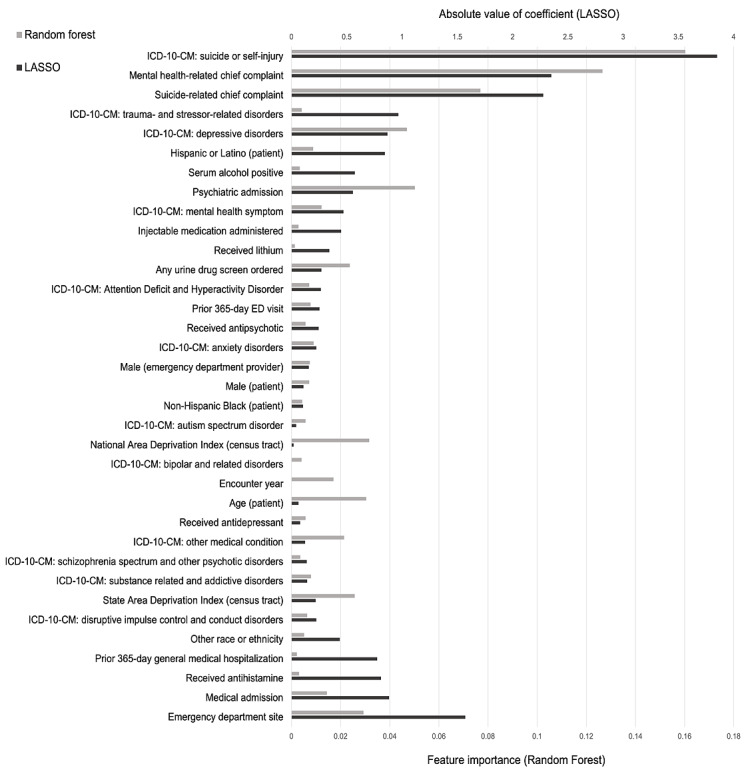
Feature importances for the classification of children’s emergency department (ED) visits for self-injurious thoughts and behaviors. The diagram depicts features (y-axis) and the absolute value of the feature importance for least absolute shrinkage and selection operator (LASSO)–penalized logistic regression (top x-axis, dark gray) and random forest (bottom x-axis, light gray). Features with nonzero feature importance are displayed and ranked in descending order such that the topmost features are those with high positive predictive performance, and the bottommost features are those with high negative predictive importance, whereas features in the middle are of the lowest importance. ADHD: attention-deficit/hyperactivity disorder; ADI: Area Deprivation Index; CC: chief complaint; ICD-10-CM: International Classification of Diseases, Tenth Revision, Clinical Modification.

The feature importances of models containing all structured data elements are presented in Figure 2, in descending order of importance, with the top predictors, including ICD-10-CM code for suicide or self-injury, mental health–related chief complaint, suicide-related chief complaint, and ICD-10-CM code for depressive disorders. Some features were identified as similarly important by both LASSO and random forest models (eg, ICD-10-CM code for depressive disorders and ICD-10-CM code for anxiety disorders), whereas other features were identified as important only in 1 model (eg, LASSO: ICD-10-CM code for trauma- and stressor-related disorders and random forest: age and national ADI).

There were significant differences in model performances by number and types of considered features. The sensitivity of detection of the machine learning models that considered all structured data elements was significantly higher than the sensitivity of detection using only suicide-related ICD-10-CM code and suicide-related chief complaint (LASSO: *χ*^2^_1_=20.2, *P*<.001 and random forest: *χ*^2^_1_=21.6, *P*<.001). However, the detection sensitivity of the models considering all structured data elements (84 features) was not significantly different from the sensitivity of the models considering a smaller number of features (25 features and 53 features): both models considering mental health–related diagnostic codes and chief complaints (LASSO: *χ*^2^_1_=0.3, *P*=.59 and random forest: *χ*^2^_1_=0.7, *P*=.39) and the models considering structured data elements other than diagnostic codes (LASSO: *χ*^2^_1_=0.6, *P*=.44 and random forest: *χ*^2^_1_=0.4, *P*=.51) did not significantly differ in sensitivity from the models considering all data elements. Fit metrics and per-fold feature importances are reported in Multimedia Appendix 8.

**Table 4:** Comparison of classifier performance of rule-based and machine learning classifiers (n=600), with machine learning classifier threshold set at 0.5.

Classifier and classification		Features	True positive, n (%)	False positive, n (%)	False negative, n (%)	True negative, n (%)	Sensitivity (95% CI)	Specificity (95% CI)	Accuracy (95% CI)
**Rule-based**									
	ICD-10-CM^a^ or CC^b,c^	2	220 (36.7)	5 (0.8)	64 (10.7)	311 (51.8)	0.77 (0.73-0.82)	0.98 (0.97-1.00)	0.89 (0.86-0.91)
**LASSO^d^**									
	Model 1^e^	34	240 (40)	28 (4.7)	44 (7.3)	288 (48)	0.85 (0.80-0.89)	0.91 (0.88-0.95)	0.88 (0.85-0.91)
	Model 2^f^	53	239 (39.8)	30 (5)	45 (7.5)	286 (47.7)	0.84 (0.79-0.89)	0.91 (0.87-0.94)	0.87 (0.85-0.90)
	Model 3^g^	84	242 (40.3)	29 (4.8)	42 (7)	287 (47.8)	0.86 (0.81-0.90)	0.91 (0.88-0.94)	0.88 (0.86-0.97)
**Random forest**									
	Model 1	34	241 (40.2)	28 (4.7)	43 (7.2)	288 (48)	0.85 (0.80-0.89)	0.91 (0.88-0.94)	0.88 (0.86-0.91)
	Model 2	53	242 (40.3)	39 (6.5)	42 (4)	277 (46.1)	0.85 (0.81-0.89)	0.88 (0.85-0.92)	0.86 (0.84-0.89)
	Model 3	84	243 (40.5)	26 (4.3)	41 (6.8)	290 (48.3)	0.86 (0.81-0.90)	0.92 (0.88-0.95)	0.88 (0.86-0.91)

^a^ICD-10-CM: International Classification of Diseases, Tenth Revision, Clinical Modification.

^b^CC: chief complaint.

^c^CC refers to suicide-related chief complaints.

^d^LASSO: least absolute shrinkage and selection operator–penalized logistic regression.

^e^Model 1 considered all mental health–related ICD-10-CM codes organized by Child and Adolescent Mental Health Disorders Classification System categories as well as suicide-related CCs and mental health–related CCs.

^f^Model 2 considered suicide-related ICD-10-CM codes and all data elements (eg, child sociodemographics, emergency department disposition, involuntary hold status, medications, and laboratory tests) except mental health–related ICD-10-CM codes.

^g^Model 3 considered all structured data elements.

## Discussion

### Principal Findings

Overall, our findings suggest that suicide-related ICD-10-CM codes and chief complaints substantially underdetect suicide-related emergency department visits and that the capacity to detect varies by sex and age group. When stratified by child demographics, suicide-related codes and chief complaints miss more male children and younger children than female children and adolescents. By contrast, machine learning–based models applied to codified health data were more sensitive in detecting suicide-related emergencies than suicide-related codes and chief complaints. When comparing machine learning–based models across health data sets with varying numbers of potential features, we found minimal differences in detection performances among models trained on all features versus those trained on mental health–related codes and chief complaints alone versus those trained on suicide-related codes and non–ICD-10-CM code–based features (eg, medications and laboratory testing). Thus, the results suggest that machine learning–based models may strengthen the sensitivity of detection of childhood-onset SITB, even when considering a focused set of potential indicators.

In this sample, nearly one-third (82/284, 28.9%) of the children presenting for suicide-related emergency care were missed by suicide-related ICD-10-CM codes, and more than half (153/284, 53.9%) of the children were missed by suicide-related chief complaints alone. Although accurate and timely detection of suicide-related emergency visits among children aligns with suicide prevention efforts by supporting tracking and rapid response to epidemiologic shifts at a population scale [38], the results of this study suggest that suicide-related codes and chief complaints alone are likely insufficient in detecting cases and potentially introduce bias regarding which children are correctly detected. The CDC National Syndromic Surveillance Program has prioritized surveillance to provide timely trend information and support public health response [26]. Using multistate public health agency reports that vary in mandates to report emergency department use for suicidal behavior, the CDC Emergency Department Surveillance of Nonfatal Suicide-Related Outcomes collects near–real-time data on nonfatal suicide-related outcomes [25]. This surveillance enabled the discovery of the rise in suicide-related emergency department visits among female adolescents aged 12 to 17 years by 50.6% during the COVID-19 pandemic [5] and provides weekly reports surveilling suicidal ideation and behavior in the state of Washington via the Rapid Health Information Network [39]. Although the surveillance of SITB is a key tool in suicide prevention, the findings of this study challenge the highly prevalent use of diagnostic codes and chief complaints as a preliminary screening tool to search for potential cases of childhood-onset SITB in clinical data sets [40-42].

This study's findings add to previously described concerns regarding the validity of suicide risk prediction models relying solely on ICD-10-CM codes to screen for the outcome of interest and discover potential antecedents [43]. The significantly poorer sensitivity of suicide-related codes and chief complaints in detecting SITB among male children and preteens and the trend (without statistical significance) toward poorer sensitivity among Black and Hispanic or Latinx children (sensitivity 0.74 vs 0.78-0.87) also raise concern that children misclassified by traditional indicators are not missed at random. The variable detection of SITB by child sociodemographics may result in biased estimates of child mental health service use and accentuate disparities; for example, bias may be introduced by unintentional omission of these children from suicide risk prediction algorithms relying on suicide-related codes and chief complaints to screen for cases. This finding builds on concern that clinical suicide risk prediction models reflect inequities in health care based on race and ethnicity and other aspects of patient identity [29].

More severe behaviors (preparatory acts and suicide attempt) were most accurately detected by requiring both suicide-related codes and chief complaints to be affirmed, whereas suicidal ideation was most accurately detected if only 1 of these (code or complaint) was required to consider the case affirmed. This is perhaps because the receipt of 2 suicide-related codified data elements may be a proxy for severity, with children with more severe behaviors receiving both data elements. The accuracy of the detection of nonsuicidal self-injurious behavior was poor compared with other SITB types, which suggests that separate phenotype definitions for types of SITB (eg, separate definitions for suicidal ideation vs preparatory acts vs suicide attempt) may produce more accurate classification than combining all SITB into a single category.

The optimal choice of detection approach may also depend on the specific use case; for instance, the results of this study suggest that suicide-related codes and chief complaints are sufficient when high specificity is important, such as flagging previous suicide-related emergencies in a patient chart. The finding that suicide-related codes and chief complaints have good specificity parallels a recent systematic assessment of self-harm coding under the ICD-10-CM in adults, which suggested that 90% of the events coded as self-harm had documentation of self-harm intent in the clinical notes [44]. In the case of a chart flag, the reduction in specificity could render a machine learning–based approach not only inconvenient but also potentially detrimental if false positives are increased. By contrast, a machine learning–based approach is more effective when maximizing sensitivity is essential, and some reduction to specificity is allowable, such as when screening data sets for potential cases. As each model generates a continuous probability of class assignment, the probability threshold may be changed depending on the use case. In uses where a high sensitivity is important to detecting all cases (eg, to not miss preteens presenting for suicide-related visits), the capacity to vary the probability threshold of classification may allow more flexibility and improved detection. These findings fit within other recent proof-of-concept applications of machine learning to classify adolescent suicidal behavior using health records, such as detection within a sample of 73 hospitalized adolescents in 1 community health system in the United States [42], a stepwise rule-based natural language processing approach evaluated on a cohort of 500 adolescents with autism spectrum disorder [8], and detection within a sample of 200 adolescents aged 11 to 17 years in contact with Child and Adolescent Mental Health Services in the United Kingdom [45].

In addition, the findings suggest that although machine learning–based approaches to detection are potentially advantageous in improving sensitivity, it may not be necessary to have access to a highly comprehensive set of data elements to meaningfully improve the sensitivity of detection. Smaller sets of mental health-related data elements, both ICD-10-CM–code based and non–ICD-10-CM–code based, performed similarly to more comprehensive data elements in the detection task. This finding aligns with work involving the phenotyping of suicidal thoughts and behaviors using discharge summaries from intensive care unit admissions in the Medical Information Mart for Intensive Care III (MIMIC-III) database and demonstrating promise of using elastic net penalized regression to detect SITB with as few as 11 features [46].

This study has several limitations. The sample is limited to a single health system in urban Los Angeles and may not generalize to less-resourced settings. The sample was also restricted to oversample case positives and individuals without missingness. Despite adjustment for selective stratification, the study sample size remained insufficient to develop and test separate machine learning–based models by sociodemographic characteristic to explore potential bias with a machine learning–based approach. The triage screening question, “Does this patient have a primary psychiatric complaint or suspicion of psychiatric illness?” was used to determine the study sample, but triage screening questions related to suicide (eg, Columbia Suicide Severity Rating Scale items) were subsequently not selected for inclusion from the classification models because questions were asked using flow sheet cascades with a high degree of nonrandom missingness of individual items. Although commonly regarded as a gold standard for classification, the manual chart review is a silver standard for truth because it ultimately depends on information documented in clinical notes that contain the biases and idiosyncrasies of the clinical documenter and may imperfectly reflect the reality of the clinical scenario. Chief complaints were documented through an electronic health record speed button and thus may not generalize to less-structured text descriptions of the presenting problem.

### Conclusions

Taken together, this study adds to existing efforts made toward developing clinical phenotypes of pediatric health conditions. Going forward, future research is needed to refine the detection of SITB across different health systems and populations, elucidate the potential advantage of including point-of-care universal suicide screening tools into phenotype detection algorithms, and determine whether including indicators of suicide-related behavior from clinical text improves detection. To achieve better integration between clinical research informatics and child mental health care, further work is needed to test the implementation of detection approaches at the point of care and assess the potential benefits of the precise identification of targets for suicide prevention interventions in children.

## Appendix

Multimedia Appendix 1 Comparison of sample-eligible children with missingness and those without missingness of diagnostic code, chief complaint, and triage screening.

Multimedia Appendix 2 Study variables (structured data elements).

Multimedia Appendix 3 Additional sample characteristics.

Multimedia Appendix 4 Laboratory testing and medications.

Multimedia Appendix 5 Sampling probability–adjusted performance of International Classification of Diseases, Tenth Revision, Clinical Modification (ICD-10-CM), code and suicide-related chief complaint in detecting cases of self-injurious thoughts and behaviors compared with that of manual chart abstraction: total sample and stratified by natal sex, age group, race, and ethnicity.

Multimedia Appendix 6 Sampling probability–adjusted performance of International Classification of Diseases, Tenth Revision, Clinical Modification (ICD-10-CM), code and suicide-related chief complaint in detecting cases of self-injurious thoughts and behaviors compared with that of manual chart abstraction: stratified by Columbia Classification Algorithm of Suicide Assessment categorization.

Multimedia Appendix 7 Comparison of classifier performance using the McNemar chi-square test.

Multimedia Appendix 8 Fit metrics and per-fold feature importances of least absolute shrinkage and selection operator (LASSO) and random forest classifiers.

## Abbreviations

ADI: Area Deprivation Index
CAMHD-CS: Child and Adolescent Mental Health Disorders Classification System
C-CASA: Columbia Classification Algorithm of Suicide Assessment
CDC: Centers for Disease Control and Prevention
FIPS: Federal Information Processing System
ICD-10-CM: International Classification of Diseases, Tenth Revision, Clinical Modification
LASSO: least absolute shrinkage and selection operator
MIMIC-III: Medical Information Mart for Intensive Care III
SITB: self-injurious thoughts and behaviors
STROBE: Strengthening the Reporting of Observational Studies in Epidemiology
