# Supplementary material for: Assessing Detection of Children With Suicide-Related Emergencies: Evaluation and Development of Computable Phenotyping Approaches
Source: JMIR Ment Health. 2023 Jul 21;10:e47084. doi: 10.2196/47084 (PMC10403798; doi:10.2196/47084)
Supplement: Multimedia Appendix 1 [file mental_v10i1e47084_app1.docx]

| **Multimedia Appendix 1.** Comparison of sample-eligible children with and without missingness on diagnostic code, chief complaint, and triage screening. | | | | |
| --- | --- | --- | --- | --- |
|  | **Category** | **With Missing** | **Without Missing** | **p-value** |
|  |  | **n (%)** | **n (%)** |  |
| Total |  | 2855 | 1713 |  |
| ICD-10 Positive |  |  |  | 0.0094 |
|  | 0 | 1866 (65.4%) | 1184 (69.1%) |  |
|  | 1 | 989 (34.6%) | 529 (30.9%) |  |
| Chief Complaint Positive |  |  |  | 0.0005 |
|  | Missing | 425 (14.9%) | 0 (0%) |  |
|  | 0 | 1903 (66.7%) | 1386 (80.9%) |  |
|  | 1 | 527 (18.5%) | 327 (19.1%) |  |
| c-SSRS Screener Positive^a^ |  |  |  | 0.0005 |
|  | Missing | 2759 (96.6%) | 0 (0%) |  |
|  | 0 | 72 (2.5%) | 1047 (61.1%) |  |
|  | 1 | 24 (0.8%) | 666 (38.9%) |  |
| Natal Sex |  |  |  |  |
|  |  |  |  | 0.8544 |
|  | 0 (Male) | 1360 (47.6%) | 811 (47.3%) |  |
|  | 1 (Female) | 1495 (52.4%) | 902 (52.7%) |  |
| Age group, yrs |  |  |  |  |
| 10-12.9 |  |  |  | 0.9386 |
|  | 0 | 2298 (80.5%) | 1377 (80.4%) |  |
|  | 1 | 557 (19.5%) | 336 (19.6%) |  |
| 13-15.9 |  |  |  | 0.1387 |
|  | 0 | 1752 (61.4%) | 1089 (63.6%) |  |
|  | 1 | 1103 (38.6%) | 624 (36.4%) |  |
| 16-17.9 |  |  |  | 0.1646 |
|  | 0 | 1660 (58.1%) | 960 (56%) |  |
|  | 1 | 1195 (41.9%) | 753 (44%) |  |
| Race/Ethnicity |  |  |  |  |
| White (non-Hispanic) |  |  |  | 0.0622 |
|  | 0 | 1364 (47.8%) | 868 (50.7%) |  |
|  | 1 | 1491 (52.2%) | 845 (49.3%) |  |
| Black (non-Hispanic) |  |  |  | 0.3949 |
|  | 0 | 2604 (91.2%) | 1549 (90.4%) |  |
|  | 1 | 251 (8.8%) | 164 (9.6%) |  |
| Asian (non-Hispanic) |  |  |  | 0.7293 |
|  | 0 | 2706 (94.8%) | 1628 (95%) |  |
|  | 1 | 149 (5.2%) | 85 (5%) |  |
| Hispanic |  |  |  | 0.0189 |
|  | 0 | 2160 (75.7%) | 1242 (72.5%) |  |
|  | 1 | 695 (24.3%) | 471 (27.5%) |  |
| Other |  |  |  | 0.3961 |
|  | 0 | 2586 (90.6%) | 1565 (91.4%) |  |
|  | 1 | 269 (9.4%) | 148 (8.6%) |  |
| Chart Review Positive |  |  |  | 0.0005 |
|  | No Chart Review | 2855 (100%) | 1113 (65%) |  |
|  | Chart Review Negative | 0 (0%) | 316 (18.4%) |  |
|  | Chart Review Positive | 0 (0%) | 284 (16.6%) |  |
