# Supplementary material for: Assessing Detection of Children With Suicide-Related Emergencies: Evaluation and Development of Computable Phenotyping Approaches
Source: JMIR Ment Health. 2023 Jul 21;10:e47084. doi: 10.2196/47084 (PMC10403798; doi:10.2196/47084)
Supplement: Multimedia Appendix 2 [file mental_v10i1e47084_app2.docx]

| **Multimedia Appendix 2.** Study Variables (Structured Data Elements) | |
| --- | --- |
| Variable name | Description |
| ip_patient_id | A de-identified, unique ID number for the individual patient (assigned by IP for coding purposes) |
| ip_enc_id | A de-identified, unique ID number for the encounter (assigned by IP for coding purposes) |
| GS Pos | Encounter positive for suicide and self-harm per manual chart review |
| Age | Patient age |
| State ADI | Neighborhood area of deprivation index (ADI) ranked scores at the state level |
| National ADI | Neighborhood area of deprivation index (ADI) ranked scores at the national level |
| Site | Academic tertiary care medical center (1); Community hospital with academic affiliation (2) |
| Medical Admission | Emergency Department visit resulted in general medical hospitalization |
| Psychiatric Admission | Emergency Department visit resulted in psychiatric hospitalization |
| Legal Status | Voluntary vs. Involuntary status (5585 hold) during Emergency Department Visit (1 if ever involuntary) |
| Chief Complaint Suicide-related | Chief complaint including key words SUICIDAL or SUICIDE ATTEMPT |
| Chief Complaint Psychiatric | Chief complaint including key words AGITATION, ALTERED MENTAL STATUS, PSYCHIATRIC EVALUATION, AGGRESSIVE BEHAVIOR, ALCOHOL INTOXICATION, DEPRESSION, AGITATION, MANIC BEHAVIOR, HALLUCINATIONS, HOMICIDAL, PANIC ATTACK, BEHAVIOR PROBLEM, ANXIETY, DELUSIONAL, EATING DISORDER, SUPPORT FOR PSYCHOLOGICAL DISTRESS, SUICIDAL, SUICIDE ATTEMPT, POISONING, DRUG OVERDOSE, INGESTION |
| Male | Natal sex male (1), Natal sex female (0) |
| Non-Hispanic White | Self-identified or caregiver-identified race/ethnicity of the child |
| Non-Hispanic Black | Self-identified or caregiver-identified race/ethnicity of the child |
| Non-Hispanic Asian | Self-identified or caregiver-identified race/ethnicity of the child |
| Hispanic | Self-identified or caregiver-identified ethnicity of the child |
| Other Race/Ethnicity | Self-identified or caregiver-identified race/ethnicity of the child |
| ICD 10 Group: Accidental or Undetermined Poisoning | ICD-10 Code - CAMHD-CS Diagnostic Group 1 |
| ICD-10 Group: ADHD | ICD-10 Code - CAMHD-CS Diagnostic Group 2 |
| ICD-10 Group: Anxiety Disorders | ICD-10 Code - CAMHD-CS Diagnostic Group 3 |
| ICD-10 Group: Autism Spectrum Disorder | ICD-10 Code - CAMHD-CS Diagnostic Group 4 |
| ICD-10 Group: Bipolar and Related Disorders | ICD-10 Code - CAMHD-CS Diagnostic Group 5 |
| ICD-10 Group: Communication Disorders | ICD-10 Code - CAMHD-CS Diagnostic Group 6 |
| ICD-10 Group: Depressive Disorders | ICD-10 Code - CAMHD-CS Diagnostic Group 7 |
| ICD-10 Group: Developmental Delay or Unspecified Neurodevelopmental Disorder | ICD-10 Code - CAMHD-CS Diagnostic Group 8 |
| ICD-10 Group: Disruptive Impulse Control and Conduct Disorders | ICD-10 Code - CAMHD-CS Diagnostic Group 9 |
| ICD-10 Group: Dissociative Disorders | ICD-10 Code - CAMHD-CS Diagnostic Group 10 |
| ICD-10 Group: Elimination Disorders | ICD-10 Code - CAMHD-CS Diagnostic Group 11 |
| ICD-10 Group: Feeding and Eating Disorders | ICD-10 Code - CAMHD-CS Diagnostic Group 12 |
| ICD-10 Group: Fetal or Newborn Damage Related to Maternal Substance Abuse | ICD-10 Code - CAMHD-CS Diagnostic Group 13 |
| ICD-10 Group: Intellectual Disability | ICD-10 Code - CAMHD-CS Diagnostic Group 14 |
| ICD-10 Group: Maternal Mental Illness or Substance Abuse | ICD-10 Code - CAMHD-CS Diagnostic Group 15 |
| ICD-10 Group: Mental Health Symptom | ICD-10 Code - CAMHD-CS Diagnostic Group 16 |
| ICD-10 Group: Miscellaneous | ICD-10 Code - CAMHD-CS Diagnostic Group 17 |
| ICD-10 Group: Motor Disorders | ICD-10 Code - CAMHD-CS Diagnostic Group 18 |
| ICD-10 Group: Neurocognitive Disorders | ICD-10 Code - CAMHD-CS Diagnostic Group 19 |
| ICD-10 Group: Obsessive Compulsive and Related Disorders | ICD-10 Code - CAMHD-CS Diagnostic Group 20 |
| ICD-10 Group: Personality Disorders | ICD-10 Code - CAMHD-CS Diagnostic Group 21 |
| ICD-10 Group: Schizophrenia Spectrum and Other Psychotic Disorders | ICD-10 Code - CAMHD-CS Diagnostic Group 22 |
| ICD-10 Group: Sexuality and Gender Identity Disorders | ICD-10 Code - CAMHD-CS Diagnostic Group 23 |
| ICD-10 Group: Sleep Wake Disorders | ICD-10 Code - CAMHD-CS Diagnostic Group 24 |
| ICD-10 Group: Somatic Symptom and Related Disorders | ICD-10 Code - CAMHD-CS Diagnostic Group 25 |
| ICD-10 Group: Specific Learning Disorders | ICD-10 Code - CAMHD-CS Diagnostic Group 26 |
| ICD-10 Group: Substance Abuse Related Medical Illness | ICD-10 Code - CAMHD-CS Diagnostic Group 27 |
| ICD-10 Group: Substance Related and Addictive Disorders | ICD-10 Code - CAMHD-CS Diagnostic Group 28 |
| ICD-10 Group: Suicide or Self-Injury | ICD-10 Code - CAMHD-CS Diagnostic Group 29, same ICD-10 codes as CDC Case Surveillance Definition (2018) |
| Trauma and Stressor-Related Disorders | ICD-10 Code - CAMHD-CS Diagnostic Group 30 |
| ICD-10 Group: Other Medical Condition | ICD-10 Code not included in CAMHD-CS |
| ICD-10 Group: Suicidal Ideation (Strict) | ICD-10 Code - Specific to suicidal ideation only (R45.851) |
| prior_90d_ed | Number of Emergency Department Visits in the last 90 days (all-cause) |
| prior_180d_ed | Number of Emergency Department Visits in the last 180 days (all-cause) |
| prior_365d_ed | Number of Emergency Department Visits in the last 365 days (all-cause) |
| prior_90d_hosp | Number of General Medical Hospitalizations in the last 90 days (all-cause) |
| prior_180d_hosp | Number of General Medical Hospitalizations in the last 180 days (all-cause) |
| prior_365d_hosp | Number of General Medical Hospitalizations in the last 365 days (all-cause) |
| prior_90d_psych | Number of Psychiatric Hospitalizations in the last 90 days (all-cause) |
| prior_180d_psych | Number of Psychiatric Hospitalizations in the last 180 days (all-cause) |
| prior_365d_psych | Number of Psychiatric Hospitalizations in the last 365 days (all-cause) |
| Antidepressants | Medication administered during ED visit. Includes amitriptyline, bupropion, citalopram, clomipramine, desvenlafaxine, doxepin, duloxetine, escitalopram, fluoxetine, fluvoxamine, imipramine, mirtazapine, paroxetine, sertraline, trazodone, venlafaxine, vilazodone, vortioxetine. |
| Antiepileptics | Medication administered during ED visit. Includes carbamazepine, divalproex, ethosuximide, gabapentin, lamotrigine, levetiracetam, oxcarbazepine, phenobarbital, phenytoin, pregabalin, topiramate, valproate, valproic. |
| Antihistamines | Medication administered during ED visit. Includes diphenhydramine, promethazine. |
| Antipsychotics | Medication administered during ED visit. Includes aripiprazole, asenapine, brexpiprazole, cariprazine, chlorpromazine, clozapine, fluphenazine, haloperidol, lurasidone, olanzapine, paliperidone, prochlorperazine, quetiapine, risperidone, ziprasidone |
| Anxiolytics | Medication administered during ED visit. Includes alprazolam, buspirone, chlordiazepoxide, clobazam, clonazepam, diazepam, hydroxyzine, lorazepam, temazepam, triazolam |
| Hypnotics and Sedatives | Medication administered during ED visit. Includes zaleplon, zolpidem, zonisamide, and melatonin |
| Lithium | Medication administered during ED visit. Includes lithium. |
| Psychostimulants, agents used for ADHD and nootropics | Medication administered during ED visit. Includes amphetamine, amphetamine_dextroamphetamine, dexmethylphenidate, dextroamphetamine, lisdexamfetamine, methylphenidate, modafinil |
| Number of medication classes | Number of medication class types administered during ED visit |
| Acetaminophen | Serum blood test ordered and collected during ED visit - resulted positive |
| Salicylates | Serum blood test ordered and collected during ED visit - resulted positive |
| Benzodiazepines | Serum blood test ordered and collected during ED visit - resulted positive |
| Tricyclics | Serum blood test ordered and collected during ED visit - resulted positive |
| Alcohol | Serum blood test ordered and collected during ED visit - resulted positive |
| Any overdose lab ordered | Serum blood test ordered and collected during ED visit (Acetaminophen, Salicylates, Benzodiazepines, Tricyclics, Alcohol)- resulted positive or negative |
| uds_amphetamine_methamphetamine | Urine drug screen ordered and collected during ED visit |
| uds_barbiturates | Urine drug screen ordered and collected during ED visit |
| uds_benzodiazepines | Urine drug screen ordered and collected during ED visit |
| uds_cannabinoids | Urine drug screen ordered and collected during ED visit |
| uds_cocaine | Urine drug screen ordered and collected during ED visit |
| uds_methadone | Urine drug screen ordered and collected during ED visit |
| uds_opiates | Urine drug screen ordered and collected during ED visit |
| uds_ethanol | Urine drug screen ordered and collected during ED visit |
| uds_oxycodone | Urine drug screen ordered and collected during ED visit |
| uds_ordered | Any urine drug screen test ordered and collected during ED visit |
| encounter_year | Year of ED visit (2015-2019) |
| provider_sex | ED physician self-identified as male (1), female (0) |
