# Supplementary material for: Assessing Detection of Children With Suicide-Related Emergencies: Evaluation and Development of Computable Phenotyping Approaches
Source: JMIR Ment Health. 2023 Jul 21;10:e47084. doi: 10.2196/47084 (PMC10403798; doi:10.2196/47084)
Supplement: Multimedia Appendix 3 [file mental_v10i1e47084_app3.docx]

| **Multimedia Appendix 3.** Additional Sample Characteristics |  |  |  |
| --- | --- | --- | --- |
| A. Emergency Department Mental Health Diagnoses of Study Population |  |  |  |
| **ED Diagnostic Code Category** | **n** | **%** |  |
| Accidental or Undetermined Poisoning | 1 | 0.2 |  |
| ADHD | 105 | 17.5 |  |
| Anxiety Disorders | 181 | 30.2 |  |
| Autism Spectrum Disorder | 59 | 9.8 |  |
| Bipolar and Related Disorders | 24 | 4.0 |  |
| Communication Disorders | 4 | 0.7 |  |
| Depressive Disorders | 221 | 36.8 |  |
| Developmental Delay or Unspecified Neurodevelopmental Disorder | 23 | 3.8 |  |
| Disruptive, Impulse Control and Conduct Disorders | 35 | 5.8 |  |
| Dissociative Disorders | 0 | 0.0 |  |
| Elimination Disorders | 2 | 0.3 |  |
| Feeding and Eating Disorders | 17 | 2.8 |  |
| Fetal or Newborn Damage Related to Maternal Substance Abuse | 1 | 0.2 |  |
| Intellectual Disability | 10 | 1.7 |  |
| Maternal Mental Illness or Substance Abuse During Preg, Delivery or Post Partum | 0 | 0.0 |  |
| Mental Health Symptom | 76 | 12.7 |  |
| Miscellaneous | 39 | 6.5 |  |
| Motor Disorders | 6 | 1.0 |  |
| Neurocognitive Disorders | 12 | 2.0 |  |
| Obsessive-Compulsive and Related Disorders | 33 | 5.5 |  |
| Non-Psychiatric / Other Medical Diagnosis | 475 | 79.2 |  |
| Personality Disorders | 4 | 0.7 |  |
| Schizophrenia Spectrum and Other Psychotic Disorders | 21 | 3.5 |  |
| Sexuality and Gender Identity Disorders | 4 | 0.7 |  |
| Sleep-Wake Disorders | 0 | 0.0 |  |
| Somatic Symptom and Related Disorders | 4 | 0.7 |  |
| Specific Learning Disorders | 6 | 1.0 |  |
| Substance Abuse-Related Medical Illness | 0 | 0.0 |  |
| Substance-Related and Addictive Disorders | 80 | 13.3 |  |
| Suicide or Self-Injury | 203 | 33.8 |  |
| Trauma and Stressor-Related Disorders | 32 | 5.3 |  |
| *Note: ICD-9 and -10 code groups from the Child and Adolescent Mental Health Disorders Classification System (CAMHD-CS)* | | |  |
|  |  |  |  |
|  |  |  |  |
| B. National Area Deprivation Index Ranking |  |  |  |
| **National ADI Ranking** | **n** | **%** |  |
| 1 - 9 | 371 | 61.8 |  |
| 10 - 19 | 94 | 15.7 |  |
| 20 - 29 | 53 | 8.8 |  |
| 30 - 39 | 31 | 5.2 |  |
| 40 - 100 | 51 | 8.5 |  |
| Missing | 56 | 9.3 |  |
| *Note: National Area Deprivation Index (ADI) Decile (California). Calculated from patient zip code. Raw score is the actual score a neighborhood receives based on the theoretical domains that the ADI measures. A decile groups the ADI scores into 10 equal sections, categorizing the individual block group/neighborhood, with those in the first percentile being the least disadvantaged, and those in the hundredth being the most. https://www.neighborhoodatlas.medicine.wisc.edu/* | | |  |
|  |  |  |  |
|  |  |  |  |
|  |  |  |  |
|  |  |  |  |
|  |  |  |  |
|  |  |  |  |
| C. Prior Acute Care Use |  |  |  |
| **Acute Care Use** | **n** | **%** |  |
| Prior ED use (≥ 1 visit) |  |  |  |
| Past 90 days | 88 | 14.7 |  |
| Past 180 days | 120 | 20.0 |  |
| Past 365 days | 159 | 26.5 |  |
| Prior Medical Hospitalization (≥ 1 visit) |  |  |  |
| Past 90 days | 20 | 3.3 |  |
| Past 180 days | 25 | 4.2 |  |
| Past 365 days | 33 | 5.5 |  |
| Prior Psychiatric Hospitalization (≥ 1 visit) |  |  |  |
| Past 90 days | 14 | 2.3 |  |
| Past 180 days | 26 | 4.3 |  |
| Past 365 days | 34 | 5.7 |  |
