# Supplementary material for: Assessing Detection of Children With Suicide-Related Emergencies: Evaluation and Development of Computable Phenotyping Approaches
Source: JMIR Ment Health. 2023 Jul 21;10:e47084. doi: 10.2196/47084 (PMC10403798; doi:10.2196/47084)
Supplement: Multimedia Appendix 4 [file mental_v10i1e47084_app4.docx]

| **Multimedia Appendix 4.** Laboratory Testing and Medications |  |  |  |  |  |  |  |
| --- | --- | --- | --- | --- | --- | --- | --- |
| A. Laboratory test results from Emergency Department Visits | |  |  |  |  |  |  |
|  | **Ordered+Resulted** | | | | **Not Ordered or Resulted** | |  |
| **Serum Tests** | **Positive** | | **Negative** | |  |  |  |
|  | **n** | **%** | **n** | **%** | **n** | **%** |  |
| Acetaminophen | 1 | 0.2 | 79 | 13.2 | 520 | 86.7 |  |
| Salicylates | 0 | 0.0 | 31 | 5.2 | 569 | 94.8 |  |
| Benzodiazepines | 0 | 0.0 | 31 | 5.2 | 569 | 94.8 |  |
| Tricyclics | 0 | 0.0 | 31 | 5.2 | 569 | 94.8 |  |
| Alcohol | 24 | 4.0 | 7 | 1.2 | 569 | 94.8 |  |
| **Urine Drug Screen** |  |  |  |  |  |  |  |
| Amphetamine/Methamphetamine | 12 | 2.0 | 143 | 23.8 | 445 | 74.2 |  |
| Barbiturates | 0 | 0.0 | 155 | 25.8 | 445 | 74.2 |  |
| Benzodiazepines | 12 | 2.0 | 143 | 23.8 | 445 | 74.2 |  |
| Cannabinoids | 31 | 5.2 | 124 | 20.7 | 445 | 74.2 |  |
| Cocaine | 2 | 0.3 | 153 | 25.5 | 445 | 74.2 |  |
| Methadone | 1 | 0.2 | 154 | 25.7 | 445 | 74.2 |  |
| Opiates | 1 | 0.2 | 154 | 25.7 | 445 | 74.2 |  |
| Ethanol | 10 | 1.7 | 145 | 24.2 | 445 | 74.2 |  |
| Oxycodone | 2 | 0.3 | 153 | 25.5 | 445 | 74.2 |  |
| *Note: For emergency department visits resulting in hospitalization, laboratory test results are only reported if ordered and collected prior to inpatient admission (≤ time of admission order). Laboratory tests that were ordered and collected in the emergency department, but resulted after inpatient admission, are included.* | | | | | | |  |
|  |  |  |  |  |  |  |  |
|  |  |  |  |  |  |  |  |
|  | | | | |  |  |  |
| B. Medications from Emergency Department Visits | | |  |  |  |  |  |

|  | |  |  |  |  |  |  |
| --- | --- | --- | --- | --- | --- | --- | --- |
| **Medication** | **n** | | **%** | **ATC Class Code** | **ATC Class Name** |  |  |
| fentanyl | 2 | | 0.3 | N01A | Anesthetic |  |  |
| ketamine | 1 | | 0.2 | N01A | Anesthetic |  |  |
| benztropine | 1 | | 0.2 | N04A | Anticholinergic agents |  |  |
| amitriptyline | 1 | | 0.2 | N06A | Antidepressants |  |  |
| bupropion | 7 | | 1.2 | N06A | Antidepressants |  |  |
| citalopram | 2 | | 0.3 | N06A | Antidepressants |  |  |
| clomipramine | 3 | | 0.5 | N06A | Antidepressants |  |  |
| desvenlafaxine | 2 | | 0.3 | N06A | Antidepressants |  |  |
| doxepin | 0 | | 0.0 | N06A | Antidepressants |  |  |
| duloxetine | 5 | | 0.8 | N06A | Antidepressants |  |  |
| escitalopram | 30 | | 5.0 | N06A | Antidepressants |  |  |
| fluoxetine | 41 | | 6.8 | N06A | Antidepressants |  |  |
| fluvoxamine | 2 | | 0.3 | N06A | Antidepressants |  |  |
| imipramine | 0 | | 0.0 | N06A | Antidepressants |  |  |
| mirtazapine | 4 | | 0.7 | N06A | Antidepressants |  |  |
| paroxetine | 3 | | 0.5 | N06A | Antidepressants |  |  |
| sertraline | 23 | | 3.8 | N06A | Antidepressants |  |  |
| trazodone | 8 | | 1.3 | N06A | Antidepressants |  |  |
| venlafaxine | 7 | | 1.2 | N06A | Antidepressants |  |  |
| vilazodone | 0 | | 0.0 | N06A | Antidepressants |  |  |
| vortioxetine | 0 | | 0.0 | N06A | Antidepressants |  |  |
| acetylcysteine | 0 | | 0.0 | V03AB | Antidotes |  |  |
| ondansetron | 38 | | 6.3 | A04 | Antiemetics |  |  |
| carbamazepine | 1 | | 0.2 | N03A | Antiepileptic |  |  |
| divalproex | 10 | | 1.7 | N03A | Antiepileptic |  |  |
| ethosuximide | 0 | | 0.0 | N03A | Antiepileptic |  |  |
| gabapentin | 8 | | 1.3 | N03A | Antiepileptic |  |  |
| lamotrigine | 14 | | 2.3 | N03A | Antiepileptic |  |  |
| levetiracetam | 0 | | 0.0 | N03A | Antiepileptic |  |  |
| oxcarbazepine | 3 | | 0.5 | N03A | Antiepileptic |  |  |
| phenobarbital | 0 | | 0.0 | N03A | Antiepileptic |  |  |
| phenytoin | 0 | | 0.0 | N03A | Antiepileptic |  |  |
| pregabalin | 0 | | 0.0 | N03A | Antiepileptic |  |  |
| topiramate | 1 | | 0.2 | N03A | Antiepileptic |  |  |
| valproate | 0 | | 0.0 | N03A | Antiepileptic |  |  |
| valproic | 0 | | 0.0 | N03A | Antiepileptic |  |  |
| diphenhydramine | 15 | | 2.5 | R06A | Antihistamine |  |  |
| diphenhydramine_hci_inj | 5 | | 0.8 | R06A | Antihistamine |  |  |
| promethazine | 0 | | 0.0 | R06A | Antihistamine |  |  |
| clonidine | 10 | | 1.7 | C02 | Antihypertensive |  |  |
| guanfacine | 15 | | 2.5 | C02 | Antihypertensive |  |  |
| prazosin | 2 | | 0.3 | C02 | Antihypertensive |  |  |
| aripiprazole | 32 | | 5.3 | N05A | Antipsychotics |  |  |
| asenapine | 0 | | 0.0 | N05A | Antipsychotics |  |  |
| brexpiprazole | 2 | | 0.3 | N05A | Antipsychotics |  |  |
| cariprazine | 0 | | 0.0 | N05A | Antipsychotics |  |  |
| chlorpromazine | 12 | | 2.0 | N05A | Antipsychotics |  |  |
| chlorpromazine_inj | 6 | | 1.0 | N05A | Antipsychotics |  |  |
| clozapine | 0 | | 0.0 | N05A | Antipsychotics |  |  |
| fluphenazine | 0 | | 0.0 | N05A | Antipsychotics |  |  |
| haloperidol | 2 | | 0.3 | N05A | Antipsychotics |  |  |
| haloperidol_inj | 2 | | 0.3 | N05A | Antipsychotics |  |  |
| lurasidone | 8 | | 1.3 | N05A | Antipsychotics |  |  |
| olanzapine | 9 | | 1.5 | N05A | Antipsychotics |  |  |
| olanzapine_for_im | 1 | | 0.2 | N05A | Antipsychotics |  |  |
| paliperidone | 0 | | 0.0 | N05A | Antipsychotics |  |  |
| prochlorperazine | 0 | | 0.0 | N05A | Antipsychotics |  |  |
| prochlorperazine_edisylate_inj | 1 | | 0.2 | N05A | Antipsychotics |  |  |
| quetiapine | 20 | | 3.3 | N05A | Antipsychotics |  |  |
| risperidone | 14 | | 2.3 | N05A | Antipsychotics |  |  |
| ziprasidone | 0 | | 0.0 | N05A | Antipsychotics |  |  |
| alprazolam | 8 | | 1.3 | N05B | Anxiolytics |  |  |
| buspirone | 6 | | 1.0 | N05B | Anxiolytics |  |  |
| chlordiazepoxide | 0 | | 0.0 | N05B | Anxiolytics |  |  |
| clobazam | 0 | | 0.0 | N05B | Anxiolytics |  |  |
| clonazepam | 13 | | 2.2 | N05B | Anxiolytics |  |  |
| diazepam | 2 | | 0.3 | N05B | Anxiolytics |  |  |
| diazepam_inj | 0 | | 0.0 | N05B | Anxiolytics |  |  |
| hydroxyzine | 11 | | 1.8 | N05B | Anxiolytics |  |  |
| hydroxyzine_hci_im | 0 | | 0.0 | N05B | Anxiolytics |  |  |
| lorazepam | 26 | | 4.3 | N05B | Anxiolytics |  |  |
| lorazepam_inj | 8 | | 1.3 | N05B | Anxiolytics |  |  |
| temazepam | 0 | | 0.0 | N05B | Anxiolytics |  |  |
| triazolam | 0 | | 0.0 | N05B | Anxiolytics |  |  |
| naltrexone | 1 | | 0.2 | N07B | Drugs used in addictive disorders |  |  |
| nicotine | 1 | | 0.2 | N07B | Drugs used in addictive disorders |  |  |
| zaleplon | 0 | | 0.0 | N05C | Hypnotics and Sedatives |  |  |
| zolpidem | 0 | | 0.0 | N05C | Hypnotics and Sedatives |  |  |
| zonisamide | 0 | | 0.0 | N05C | Hypnotics and Sedatives |  |  |
| melatonin | 10 | | 1.7 | N05C | Hypnotics and Sedatives |  |  |
| lithium | 11 | | 1.8 | N05AN | Lithium |  |  |
| methadone | 0 | | 0.0 | N02A | Opioids |  |  |
| morphine | 5 | | 0.8 | N02A | Opioids |  |  |
| naloxone | 0 | | 0.0 | N02A | Opioids |  |  |
| oxycodone | 4 | | 0.7 | N02A | Opioids |  |  |
| oxycodone_acetaminophen | 0 | | 0.0 | N02A | Opioids |  |  |
| tramadol | 1 | | 0.2 | N02A | Opioids |  |  |
| amphetamine | 1 | | 0.2 | N06B | Psychostimulants, agents used for ADHD and nootropics |  |  |
| amphetamine_dextroamphetamine | 7 | | 1.2 | N06B | Psychostimulants, agents used for ADHD and nootropics |  |  |
| dexmethylphenidate | 5 | | 0.8 | N06B | Psychostimulants, agents used for ADHD and nootropics |  |  |
| dextroamphetamine | 1 | | 0.2 | N06B | Psychostimulants, agents used for ADHD and nootropics |  |  |
| lisdexamfetamine | 8 | | 1.3 | N06B | Psychostimulants, agents used for ADHD and nootropics |  |  |
| methylphenidate | 6 | | 1.0 | N06B | Psychostimulants, agents used for ADHD and nootropics |  |  |
| modafinil | 0 | | 0.0 | N06B | Psychostimulants, agents used for ADHD and nootropics |  |  |
| fexofenadine | 0 | | 0.0 | R01BA | Sympathomimetic |  |  |
| levothyroxine | 1 | | 0.2 | H03 | Thyroid therapy |  |  |
| l_methylfolate | 1 | | 0.2 | B03B | Vitamine B12 and folic acid |  |  |
| *Note: Oral formulation unless specified. For emergency department visits resulting in hospitalization, medications are only reported if ordered/taken prior to inpatient admission (≤ time of admission order).* | | | | | |  |  |
|  |  |  |  |  |  |  |  |
|  |  | |  |  |  |  |  |

| C. Medication Class from Emergency Department Visits | |  |  |  |  |  |  |  |
| --- | --- | --- | --- | --- | --- | --- | --- | --- |
| **Class** | **n** | | **%** |  |  |  |  |  |
| Antidepressants (N06A) | 138 | | 23.0 |  |  |  |  |  |
| Antiepileptics (N03A) | 37 | | 6.2 |  |  |  |  |  |
| Antihistamines (R06A) | 20 | | 3.3 |  |  |  |  |  |
| Antipsychotics (N05A) | 109 | | 18.2 |  |  |  |  |  |
| Anxiolytics (N05B) | 74 | | 12.3 |  |  |  |  |  |
| Hypnotics and Sedatives (N05C) | 10 | | 1.7 |  |  |  |  |  |
| Lithium (N05A) | 11 | | 1.8 |  |  |  |  |  |
| Psychostimulants, agents used for ADHD (N06B) | 28 | | 4.7 |  |  |  |  |  |
| Injectables | 23 | | 3.8 |  |  |  |  |  |
| *Note: Most antiepileptics (33/37, 89%) were lamotrigine (n=14), divalproex (n=10) and gabapentin (n=9)* | | | | |  |  |  |  |
