# Supplementary material for: Assessing Detection of Children With Suicide-Related Emergencies: Evaluation and Development of Computable Phenotyping Approaches
Source: JMIR Ment Health. 2023 Jul 21;10:e47084. doi: 10.2196/47084 (PMC10403798; doi:10.2196/47084)
Supplement: Multimedia Appendix 5 [file mental_v10i1e47084_app5.docx]

| **Multimedia Appendix 5** Sampling Probability Adjusted Performance of ICD-10 Code and Suicide-Related Chief Complaint in Detecting Cases of Self-Injurious Thoughts and Behaviors Compared with Manual Chart Abstraction: Total Sample and Stratified by Natal Sex, Age Group, Race, and Ethnicity | | | | | | | | | | | | | | | | |  |
| --- | --- | --- | --- | --- | --- | --- | --- | --- | --- | --- | --- | --- | --- | --- | --- | --- | --- |
|  |  |  |  |  |  | **Sensitivity** | | |  | **Specificity** | | |  | **Accuracy** | | |  |
|  |  |  |  |  |  |  | **95% CI** | |  |  | **95% CI** | |  |  | **95% CI** | |  |
| All | **True +** | **False +** | **False -** | **True -** |  |  | **UL** | **LL** |  |  | **UL** | **LL** |  |  | **UL** | **LL** |  |
| CC | 129 | 2 | 155 | 314 |  | 0.44 | 0.42 | 0.46 |  | 1.00 | 0.99 | 1.00 |  | 0.76 | 0.73 | 0.78 |  |
| ICD | 199 | 4 | 85 | 312 |  | 0.71 | 0.67 | 0.74 |  | 0.99 | 0.98 | 1.00 |  | 0.87 | 0.85 | 0.89 |  |
| CC +/- ICD | 220 | 5 | 64 | 311 |  | 0.77 | 0.73 | 0.80 |  | 0.99 | 0.98 | 1.00 |  | 0.89 | 0.87 | 0.91 |  |
| CC + ICD | 108 | 1 | 176 | 315 |  | 0.38 | 0.36 | 0.40 |  | 1.00 | 0.99 | 1.00 |  | 0.73 | 0.71 | 0.76 |  |
| Males |  |  |  |  |  |  |  |  |  |  |  |  |  |  |  |  |  |
| CC | 52 | 0 | 71 | 153 |  | 0.37 | 0.33 | 0.40 |  | 1.00 | 0.99 | 1.00 |  | 0.75 | 0.71 | 0.79 |  |
| ICD | 78 | 0 | 45 | 153 |  | 0.61 | 0.55 | 0.67 |  | 1.00 | 0.99 | 1.00 |  | 0.85 | 0.81 | 0.88 |  |
| CC +/- ICD | 85 | 0 | 38 | 153 |  | 0.65 | 0.59 | 0.71 |  | 1.00 | 0.99 | 1.00 |  | 0.86 | 0.82 | 0.90 |  |
| CC + ICD | 45 | 0 | 78 | 153 |  | 0.33 | 0.30 | 0.36 |  | 1.00 | 0.99 | 1.00 |  | 0.73 | 0.69 | 0.77 |  |
| Females |  |  |  |  |  |  |  |  |  |  |  |  |  |  |  |  |  |
| CC | 77 | 2 | 84 | 161 |  | 0.49 | 0.46 | 0.52 |  | 0.99 | 0.98 | 1.00 |  | 0.76 | 0.74 | 0.79 |  |
| ICD | 121 | 4 | 40 | 159 |  | 0.78 | 0.74 | 0.82 |  | 0.98 | 0.96 | 1.00 |  | 0.89 | 0.86 | 0.91 |  |
| CC +/- ICD | 135 | 5 | 26 | 158 |  | 0.85 | 0.81 | 0.90 |  | 0.98 | 0.95 | 1.00 |  | 0.92 | 0.89 | 0.95 |  |
| CC + ICD | 63 | 1 | 98 | 162 |  | 0.42 | 0.39 | 0.44 |  | 0.99 | 0.98 | 1.00 |  | 0.73 | 0.70 | 0.76 |  |
| 10-12.9 y |  |  |  |  |  |  |  |  |  |  |  |  |  |  |  |  |  |
| CC | 25 | 0 | 34 | 56 |  | 0.38 | 0.32 | 0.43 |  | 1.00 | 0.98 | 1.00 |  | 0.73 | 0.67 | 0.79 |  |
| ICD | 33 | 0 | 26 | 56 |  | 0.56 | 0.48 | 0.64 |  | 1.00 | 0.96 | 1.00 |  | 0.81 | 0.75 | 0.87 |  |
| CC +/- ICD | 39 | 0 | 20 | 56 |  | 0.63 | 0.54 | 0.72 |  | 1.00 | 0.96 | 1.00 |  | 0.84 | 0.78 | 0.90 |  |
| CC + ICD | 19 | 0 | 40 | 56 |  | 0.31 | 0.26 | 0.36 |  | 1.00 | 0.98 | 1.00 |  | 0.70 | 0.64 | 0.77 |  |
| 13-15.9 y |  |  |  |  |  |  |  |  |  |  |  |  |  |  |  |  |  |
| CC | 57 | 1 | 59 | 98 |  | 0.48 | 0.45 | 0.52 |  | 0.99 | 0.98 | 1.00 |  | 0.75 | 0.71 | 0.78 |  |
| ICD | 90 | 1 | 26 | 98 |  | 0.80 | 0.75 | 0.85 |  | 0.99 | 0.98 | 1.00 |  | 0.90 | 0.87 | 0.93 |  |
| CC +/- ICD | 100 | 2 | 16 | 97 |  | 0.86 | 0.81 | 0.92 |  | 0.99 | 0.97 | 1.00 |  | 0.93 | 0.89 | 0.96 |  |
| CC + ICD | 47 | 0 | 69 | 99 |  | 0.42 | 0.39 | 0.46 |  | 1.00 | 0.98 | 1.00 |  | 0.72 | 0.68 | 0.76 |  |
| 16-17.9 y |  |  |  |  |  |  |  |  |  |  |  |  |  |  |  |  |  |
| CC | 47 | 1 | 62 | 160 |  | 0.42 | 0.38 | 0.46 |  | 0.99 | 0.98 | 1.00 |  | 0.78 | 0.74 | 0.81 |  |
| ICD | 76 | 3 | 33 | 158 |  | 0.68 | 0.62 | 0.74 |  | 0.98 | 0.97 | 1.00 |  | 0.87 | 0.83 | 0.90 |  |
| CC +/- ICD | 81 | 3 | 28 | 158 |  | 0.73 | 0.67 | 0.79 |  | 0.98 | 0.96 | 1.00 |  | 0.89 | 0.85 | 0.92 |  |
| CC + ICD | 42 | 1 | 67 | 160 |  | 0.37 | 0.34 | 0.41 |  | 0.99 | 0.98 | 1.00 |  | 0.76 | 0.72 | 0.79 |  |
| White, non-Hispanic |  |  |  |  |  |  |  |  |  |  |  |  |  |  |  |  |  |
| CC | 68 | 1 | 80 | 136 |  | 0.43 | 0.39 | 0.46 |  | 0.99 | 0.98 | 1.00 |  | 0.74 | 0.70 | 0.77 |  |
| ICD | 106 | 2 | 42 | 135 |  | 0.73 | 0.67 | 0.78 |  | 0.99 | 0.97 | 1.00 |  | 0.87 | 0.83 | 0.90 |  |
| CC +/- ICD | 116 | 2 | 32 | 135 |  | 0.78 | 0.72 | 0.83 |  | 0.99 | 0.97 | 1.00 |  | 0.89 | 0.86 | 0.92 |  |
| CC + ICD | 58 | 1 | 90 | 136 |  | 0.38 | 0.35 | 0.41 |  | 0.99 | 0.98 | 1.00 |  | 0.71 | 0.68 | 0.75 |  |
| Hispanic or Latino |  |  |  |  |  |  |  |  |  |  |  |  |  |  |  |  |  |
| CC | 32 | 1 | 37 | 91 |  | 0.43 | 0.39 | 0.48 |  | 0.99 | 0.97 | 1.00 |  | 0.77 | 0.73 | 0.81 |  |
| ICD | 44 | 1 | 25 | 91 |  | 0.63 | 0.56 | 0.69 |  | 0.99 | 0.98 | 1.00 |  | 0.85 | 0.81 | 0.89 |  |
| CC +/- ICD | 51 | 2 | 18 | 90 |  | 0.71 | 0.64 | 0.78 |  | 0.98 | 0.96 | 1.00 |  | 0.87 | 0.83 | 0.92 |  |
| CC + ICD | 25 | 0 | 44 | 92 |  | 0.35 | 0.30 | 0.40 |  | 1.00 | 0.97 | 1.00 |  | 0.74 | 0.69 | 0.79 |  |
| Black, non-Hispanic |  |  |  |  |  |  |  |  |  |  |  |  |  |  |  |  |  |
| CC | 12 | 0 | 19 | 30 |  | 0.43 | 0.35 | 0.51 |  | 1.00 | 0.94 | 1.00 |  | 0.73 | 0.64 | 0.82 |  |
| ICD | 21 | 0 | 10 | 30 |  | 0.66 | 0.55 | 0.78 |  | 1.00 | 0.94 | 1.00 |  | 0.84 | 0.76 | 0.93 |  |
| CC +/- ICD | 23 | 0 | 8 | 30 |  | 0.72 | 0.59 | 0.84 |  | 1.00 | 0.94 | 1.00 |  | 0.87 | 0.78 | 0.95 |  |
| CC + ICD | 10 | 0 | 21 | 30 |  | 0.38 | 0.30 | 0.45 |  | 1.00 | 0.94 | 1.00 |  | 0.71 | 0.62 | 0.79 |  |
| Other^a^ |  |  |  |  |  |  |  |  |  |  |  |  |  |  |  |  |  |
| CC | 12 | 0 | 9 | 37 |  | 0.51 | 0.39 | 0.63 |  | 1.00 | 0.96 | 1.00 |  | 0.82 | 0.74 | 0.91 |  |
| ICD | 16 | 1 | 5 | 36 |  | 0.77 | 0.63 | 0.91 |  | 0.98 | 0.95 | 1.00 |  | 0.91 | 0.84 | 0.97 |  |
| CC +/- ICD | 17 | 1 | 4 | 36 |  | 0.81 | 0.67 | 0.96 |  | 0.97 | 0.94 | 1.00 |  | 0.92 | 0.85 | 0.98 |  |
| CC + ICD | 11 | 0 | 10 | 37 |  | 0.47 | 0.36 | 0.58 |  | 1.00 | 0.96 | 1.00 |  | 0.81 | 0.73 | 0.89 |  |
| Asian, non-Hispanic |  |  |  |  |  |  |  |  |  |  |  |  |  |  |  |  |  |
| CC | 5 | 0 | 10 | 20 |  | 0.45 | 0.32 | 0.58 |  | 1.00 | 0.90 | 1.00 |  | 0.77 | 0.66 | 0.88 |  |
| ICD | 12 | 0 | 3 | 20 |  | 0.83 | 0.65 | 1.00 |  | 1.00 | 0.89 | 1.00 |  | 0.93 | 0.82 | 1.00 |  |
| CC +/- ICD | 13 | 0 | 2 | 20 |  | 0.86 | 0.68 | 1.00 |  | 1.00 | 0.89 | 1.00 |  | 0.94 | 0.83 | 1.00 |  |
| CC + ICD | 4 | 0 | 11 | 20 |  | 0.41 | 0.28 | 0.53 |  | 1.00 | 0.90 | 1.00 |  | 0.76 | 0.65 | 0.87 |  |
| *Note: a. Other = American Indian or Alaska Native, multiple races, "NA", other, patient refused, and unknown; CC = Suicide-related Chief Complaint, ICD = ICD-10 code for self-injurious thoughts and behaviors as defined by the Centers for Disease Control and Prevention Case Surveillance Definition; 95%CI refers to the 95% confidence interval lower (LL) and upper (UL) limits.* | | | | | | | | | | | | | | | | |  |
|  |  |  |  |  |  |  |  |  |  |  |  |  |  |  |  |  |  |
|  |  |  |  |  |  |  |  |  |  |  |  |  |  |  |  |  |  |
|  |  |  |  |  |  |  |  |  |  |  |  |  |  |  |  |  |  |
|  |  |  |  |  |  |  |  |  |  |  |  |  |  |  |  |  |  |
