# Supplementary material for: Assessing Detection of Children With Suicide-Related Emergencies: Evaluation and Development of Computable Phenotyping Approaches
Source: JMIR Ment Health. 2023 Jul 21;10:e47084. doi: 10.2196/47084 (PMC10403798; doi:10.2196/47084)
Supplement: Multimedia Appendix 6 [file mental_v10i1e47084_app6.docx]

| **Multimedia Appendix 6.** Sampling Probability Adjusted Performance of ICD-10 Code and Suicide-Related Chief Complaint in Detecting Cases of Self-Injurious Thoughts and Behaviors Compared with Manual Chart Abstraction: Stratified by Columbia Classification Algorithm of Suicide Assessment categorization. | | | | | | | | | | | | | | | | | |  |
| --- | --- | --- | --- | --- | --- | --- | --- | --- | --- | --- | --- | --- | --- | --- | --- | --- | --- | --- |
|  |  |  |  |  |  |  | **Sensitivity** | | |  | **Specificity** | | |  | **Accuracy** | | |  |
|  |  |  |  |  |  |  |  | **95% CI** | |  |  | **95% CI** | |  |  | **95% CI** | |  |
| **n = 600** |  | **True +** | **False +** | **False -** | **True -** |  |  | **UL** | **LL** |  |  | **UL** | **LL** |  |  | **UL** | **LL** |  |
| Suicidal ideation |  |  |  |  |  |  |  |  |  |  |  |  |  |  |  |  |  |  |
| CC |  | 73 | 58 | 71 | 398 |  | 0.50 | 0.56 | 0.44 |  | 0.89 | 0.91 | 0.87 |  | 0.81 | 0.83 | 0.78 |  |
| ICD |  | 105 | 98 | 39 | 358 |  | 0.75 | 0.80 | 0.69 |  | 0.81 | 0.83 | 0.79 |  | 0.80 | 0.82 | 0.77 |  |
| CC +/- ICD |  | 118 | 97 | 26 | 359 |  | 0.82 | 0.88 | 0.76 |  | 0.80 | 0.82 | 0.77 |  | 0.80 | 0.83 | 0.78 |  |
| CC + ICD |  | 60 | 49 | 84 | 407 |  | 0.42 | 0.48 | 0.36 |  | 0.91 | 0.92 | 0.89 |  | 0.80 | 0.83 | 0.78 |  |
| Preparatory acts |  |  |  |  |  |  |  |  |  |  |  |  |  |  |  |  |  |  |
| CC |  | 23 | 108 | 22 | 447 |  | 0.50 | 0.63 | 0.36 |  | 0.83 | 0.84 | 0.82 |  | 0.81 | 0.83 | 0.79 |  |
| ICD |  | 35 | 168 | 10 | 387 |  | 0.78 | 0.90 | 0.67 |  | 0.73 | 0.74 | 0.71 |  | 0.73 | 0.75 | 0.71 |  |
| CC +/- ICD |  | 36 | 179 | 9 | 376 |  | 0.82 | 0.93 | 0.71 |  | 0.70 | 0.71 | 0.69 |  | 0.71 | 0.73 | 0.69 |  |
| CC + ICD |  | 20 | 89 | 33 | 458 |  | 0.47 | 0.60 | 0.33 |  | 0.86 | 0.87 | 0.85 |  | 0.83 | 0.85 | 0.81 |  |
| Suicide attempt |  |  |  |  |  |  |  |  |  |  |  |  |  |  |  |  |  |  |
| CC |  | 22 | 109 | 31 | 438 |  | 0.39 | 0.51 | 0.27 |  | 0.83 | 0.84 | 0.82 |  | 0.79 | 0.81 | 0.77 |  |
| ICD |  | 42 | 161 | 11 | 386 |  | 0.77 | 0.89 | 0.66 |  | 0.73 | 0.75 | 0.72 |  | 0.74 | 0.76 | 0.72 |  |
| CC +/- ICD |  | 36 | 179 | 17 | 368 |  | 0.80 | 0.91 | 0.69 |  | 0.71 | 0.72 | 0.69 |  | 0.71 | 0.73 | 0.69 |  |
| CC + ICD |  | 21 | 88 | 24 | 466 |  | 0.37 | 0.48 | 0.25 |  | 0.85 | 0.86 | 0.84 |  | 0.81 | 0.83 | 0.79 |  |
| Non-suicidal self-injurious behavior |  |  |  |  |  |  |  |  |  |  |  |  |  |  |  |  |  |  |
| CC |  | 47 | 84 | 62 | 407 |  | 0.42 | 0.50 | 0.35 |  | 0.85 | 0.87 | 0.84 |  | 0.78 | 0.81 | 0.76 |  |
| ICD |  | 74 | 129 | 35 | 362 |  | 0.69 | 0.77 | 0.62 |  | 0.77 | 0.78 | 0.75 |  | 0.75 | 0.78 | 0.73 |  |
| CC +/- ICD |  | 81 | 134 | 28 | 357 |  | 0.76 | 0.83 | 0.69 |  | 0.75 | 0.77 | 0.73 |  | 0.75 | 0.77 | 0.72 |  |
| CC + ICD |  | 37 | 72 | 71 | 420 |  | 0.35 | 0.43 | 0.28 |  | 0.87 | 0.89 | 0.86 |  | 0.79 | 0.81 | 0.76 |  |
| *Note: CC = Suicide-related Chief Complaint, ICD = ICD-10 code for self-injurious thoughts and behaviors as defined by the Centers for Disease Control and Prevention Case Surveillance Definition; 95%CI = 95% confidence interval lower (LL) and upper (UL) limits. Other race/ethnicity = multiple races, "NA", other, patient refused, and unknown.* | | | | | | | | | | | | | | | | | |  |
|  |  |  |  |  |  |  |  |  |  |  |  |  |  |  |  |  |  |  |
|  |  |  |  |  |  |  |  |  |  |  |  |  |  |  |  |  |  |  |
