# Supplementary material for: Assessing Detection of Children With Suicide-Related Emergencies: Evaluation and Development of Computable Phenotyping Approaches
Source: JMIR Ment Health. 2023 Jul 21;10:e47084. doi: 10.2196/47084 (PMC10403798; doi:10.2196/47084)
Supplement: Multimedia Appendix 7 [file mental_v10i1e47084_app7.docx]

| **Multimedia Appendix 7.** Comparison of Classifier Performance using McNemar χ2 Test for Full-feature Models | | | | | | | |
| --- | --- | --- | --- | --- | --- | --- | --- |
|  |  | **Sensitivity** | | **Specificity** | | **Accuracy** | |
| **Test** | **Comparator** | **χ2** | **p-value** | **χ2** | **p-value** | **χ2** | **p-value** |
| Lasso | Random Forest | 0.600 | 0.439 | 1.000 | 0.317 | 0.032 | 0.857 |
| ICD/CC | Lasso | 20.167 | 0.000 | 24.000 | 0.000 | 0.083 | 0.773 |
| ICD/CC | Random Forest | 21.552 | 0.000 | 28.000 | 0.000 | 0.158 | 0.691 |
