# Supplementary material for: Assessing Detection of Children With Suicide-Related Emergencies: Evaluation and Development of Computable Phenotyping Approaches
Source: JMIR Ment Health. 2023 Jul 21;10:e47084. doi: 10.2196/47084 (PMC10403798; doi:10.2196/47084)
Supplement: Multimedia Appendix 8 [file mental_v10i1e47084_app8.docx]

| **Multimedia Appendix 8**. Machine Learning Classifier Model Output: Fit Metrics and Feature Importance | | | | | | | | | | | |
| --- | --- | --- | --- | --- | --- | --- | --- | --- | --- | --- | --- |
| 1. Model 1: Lasso-penalized logistic regression |  |  |  |  |  |  |  |  |  |  |  |
|  | TP | FN | TN | FP |  |  |  |  |  |  |  |
| Count | 240 | 44 | 288 | 28 |  |  |  |  |  |  |  |
| Proportion of total N | 0.40 | 0.07 | 0.48 | 0.05 |  |  |  |  |  |  |  |
|  |  |  |  |  |  |  |  |  |  |  |  |
|  | Sensitivity | Specificity | PPV | NPV | Accuracy | F-Statistic (weighted avg) | ROC-AUC | PR-AUC |  |  |  |
| Mean | 0.85 | 0.91 | 0.90 | 0.87 | 0.88 | 0.88 | 0.96 | 0.96 |  |  |  |
| SD | 0.06 | 0.06 | 0.07 | 0.07 | 0.03 | 0.03 | 0.02 | 0.03 |  |  |  |
| 95%CI upper | 0.89 | 0.95 | 0.92 | 0.89 | 0.91 | 0.91 | 0.98 | 0.98 |  |  |  |
| 95%CI lower | 0.80 | 0.88 | 0.87 | 0.84 | 0.85 | 0.85 | 0.95 | 0.95 |  |  |  |
| Max | 0.94 | 1.00 | 1.00 | 0.94 | 0.93 | 0.93 | 0.99 | 0.99 |  |  |  |
| Min | 0.76 | 0.83 | 0.79 | 0.71 | 0.85 | 0.85 | 0.93 | 0.90 |  |  |  |
|  |  |  |  |  |  |  |  |  |  |  |  |
|  | fold 1 | fold 2 | fold 3 | fold 4 | fold 5 | fold 6 | fold 7 | fold 8 | fold 9 | fold 10 | Median |
| Iterations | 19.00 | 17.00 | 17.00 | 14.00 | 19.00 | 18.00 | 16.00 | 15.00 | 21.00 | 16.00 | 17.00 |
| Intercepts | -3.14 | -3.07 | -3.06 | -3.24 | -3.31 | -3.19 | -3.08 | -3.15 | -3.21 | -3.48 | -3.17 |
| Features |  |  |  |  |  |  |  |  |  |  |  |
| Suicide-related CC | 2.33 | 2.71 | 2.37 | 2.45 | 2.16 | 2.34 | 2.24 | 2.41 | 2.09 | 2.60 | 2.35 |
| Mental Health-related CC | 2.56 | 2.45 | 2.32 | 2.41 | 2.35 | 2.46 | 2.41 | 2.26 | 2.48 | 2.64 | 2.43 |
| ICD 10 Group: Accidental or Undetermined Poisoning | 0.00 | 0.00 | 0.00 | 0.00 | 0.00 | 0.00 | 0.00 | 0.00 | 0.00 | 0.00 | 0.00 |
| ICD-10 Group: ADHD | 0.54 | 0.31 | 0.52 | 0.28 | 0.31 | 0.40 | 0.23 | 0.50 | 0.54 | 0.60 | 0.45 |
| ICD-10 Group: Anxiety Disorders | 0.00 | 0.15 | 0.00 | 0.19 | 0.20 | 0.13 | 0.20 | 0.19 | 0.00 | 0.21 | 0.17 |
| ICD-10 Group: Autism Spectrum Disorder | 0.31 | 0.07 | 0.24 | 0.37 | 0.53 | 0.43 | 0.26 | 0.30 | 0.16 | 0.29 | 0.30 |
| ICD-10 Group: Bipolar and Related Disorders | 0.00 | 0.00 | 0.05 | 0.41 | 0.42 | 0.32 | 0.44 | 0.00 | 0.43 | 0.14 | 0.23 |
| ICD-10 Group: Communication Disorders | 0.00 | 0.00 | 0.00 | 0.00 | 0.00 | 0.00 | 0.00 | 0.00 | 0.00 | 0.00 | 0.00 |
| ICD-10: Depressive Disorders | 0.93 | 0.98 | 0.93 | 0.90 | 0.92 | 0.83 | 0.94 | 0.68 | 0.70 | 1.02 | 0.93 |
| ICD-10 Group: Developmental Delay or Unspecified Neurodevelopmental Disorder | 0.00 | 0.00 | 0.00 | 0.00 | 0.00 | 0.00 | 0.00 | 0.00 | 0.00 | 0.00 | 0.00 |
| ICD-10 Group: Disruptive Impulse Control and Conduct Disorders | -0.16 | -0.21 | -0.08 | -0.17 | 0.00 | 0.00 | 0.00 | 0.00 | -0.12 | -0.21 | -0.10 |
| ICD-10 Group: Dissociative Disorders | 0.00 | 0.00 | 0.00 | 0.00 | 0.00 | 0.00 | 0.00 | 0.00 | 0.00 | 0.00 | 0.00 |
| ICD-10 Group: Elimination Disorders | 0.00 | 0.00 | 0.00 | 0.00 | 0.00 | 0.00 | 0.00 | 0.00 | 0.00 | 0.00 | 0.00 |
| ICD-10 Group: Feeding and Eating Disorders | -0.66 | 0.00 | -0.57 | -0.74 | -0.76 | -0.56 | -0.50 | -0.54 | 0.00 | -0.93 | -0.56 |
| ICD-10 Group: Fetal or Newborn Damage Related to Maternal Substance Abuse | 0.00 | 0.00 | 0.00 | 0.00 | 0.00 | 0.00 | 0.00 | 0.00 | 0.00 | 0.00 | 0.00 |
| ICD-10 Group: Intellectual Disability | 0.00 | 0.00 | 0.00 | 0.00 | 0.00 | 0.00 | 0.00 | 0.00 | 0.00 | 0.00 | 0.00 |
| ICD-10 Group: Maternal Mental Illness or Substance Abuse | 0.00 | 0.00 | 0.00 | 0.00 | 0.00 | 0.00 | 0.00 | 0.00 | 0.00 | 0.00 | 0.00 |
| ICD-10 Group: Mental Health Symptom | 0.76 | 0.83 | 0.85 | 0.80 | 1.00 | 0.80 | 0.71 | 1.03 | 0.64 | 0.85 | 0.82 |
| ICD-10 Group: Miscellaneous | 0.00 | 0.00 | 0.13 | 0.00 | 0.00 | 0.07 | 0.00 | 0.00 | 0.00 | 0.00 | 0.00 |
| ICD-10 Group: Motor Disorders | 0.00 | 0.00 | 0.00 | 0.00 | 0.00 | 0.00 | 0.00 | 0.00 | 0.00 | 0.00 | 0.00 |
| ICD-10 Group: Neurocognitive Disorders | 0.00 | 0.00 | 0.00 | 0.00 | 0.00 | 0.00 | 0.00 | 0.00 | 0.00 | 0.00 | 0.00 |
| ICD-10 Group: Obsessive Compulsive and Related Disorders | 0.00 | 0.00 | -0.11 | 0.00 | 0.00 | 0.00 | 0.00 | 0.00 | 0.00 | 0.00 | 0.00 |
| ICD-10 Group: Other Medical Condition | -0.50 | -0.48 | -0.48 | -0.42 | -0.46 | -0.46 | -0.44 | -0.31 | -0.29 | -0.36 | -0.45 |
| ICD-10 Group: Personality Disorders | 0.00 | 0.00 | 0.00 | 0.00 | 0.00 | 0.00 | 0.00 | 0.00 | 0.00 | 0.00 | 0.00 |
| ICD-10 Group: Schizophrenia Spectrum and Other Psychotic Disorders | 0.00 | 0.00 | 0.00 | 0.00 | 0.00 | -0.25 | -0.25 | 0.00 | -0.16 | -0.07 | 0.00 |
| ICD-10 Group: Sexuality and Gender Identity Disorders | 0.00 | 0.00 | 0.00 | 0.00 | 0.00 | 0.00 | 0.00 | 0.00 | 0.00 | 0.00 | 0.00 |
| ICD-10 Group: Sleep Wake Disorders | 0.00 | 0.00 | 0.00 | 0.00 | 0.00 | 0.00 | 0.00 | 0.00 | 0.00 | 0.00 | 0.00 |
| ICD-10 Group: Somatic Symptom and Related Disorders | 0.00 | 0.00 | 0.00 | 0.00 | 0.00 | 0.00 | 0.00 | 0.00 | 0.00 | 0.00 | 0.00 |
| ICD-10 Group: Specific Learning Disorders | 0.00 | 0.00 | 0.00 | 0.00 | 0.00 | 0.00 | 0.00 | 0.00 | 0.00 | 0.00 | 0.00 |
| ICD-10 Group: Substance Abuse Related Medical Illness | 0.00 | 0.00 | 0.00 | 0.00 | 0.00 | 0.00 | 0.00 | 0.00 | 0.00 | 0.00 | 0.00 |
| ICD-10 Group: Substance Related and Addictive Disorders | -0.58 | -0.68 | -0.62 | -0.58 | -0.82 | -0.56 | -0.65 | -0.52 | -0.54 | -0.67 | -0.60 |
| ICD-10: Suicide or Self-Injury | 3.74 | 3.60 | 3.78 | 3.81 | 4.03 | 3.86 | 3.89 | 4.02 | 3.79 | 4.39 | 3.84 |
| ICD-10 Group: Suicidal Ideation (Strict) | 0.00 | 0.00 | 0.00 | 0.00 | 0.00 | 0.00 | 0.00 | 0.00 | 0.00 | 0.00 | 0.00 |
| ICD-10 Group: Trauma- and Stressor-related Disorders | 0.31 | 0.35 | 0.64 | 0.63 | 0.69 | 0.58 | 0.09 | 0.26 | 0.94 | 0.50 | 0.54 |
|  |  |  |  |  |  |  |  |  |  |  |  |
| 2. Model 2: Lasso-penalized logistic regression |  |  |  |  |  |  |  |  |  |  |  |
|  | TP | FN | TN | FP |  |  |  |  |  |  |  |
| Count | 239 | 45 | 286 | 30 |  |  |  |  |  |  |  |
| Proportion of total N | 0.40 | 0.08 | 0.48 | 0.05 |  |  |  |  |  |  |  |
|  |  |  |  |  |  |  |  |  |  |  |  |
|  | Sensitivity | Specificity | PPV | NPV | Accuracy | F-Statistic (weighted avg) | ROC-AUC | PR-AUC |  |  |  |
| Mean | 0.84 | 0.91 | 0.89 | 0.86 | 0.88 | 0.88 | 0.97 | 0.97 |  |  |  |
| SD | 0.06 | 0.04 | 0.06 | 0.07 | 0.03 | 0.03 | 0.01 | 0.02 |  |  |  |
| 95%CI upper | 0.89 | 0.94 | 0.91 | 0.89 | 0.90 | 0.90 | 0.98 | 0.98 |  |  |  |
| 95%CI lower | 0.80 | 0.87 | 0.86 | 0.83 | 0.85 | 0.85 | 0.95 | 0.95 |  |  |  |
| Max | 0.94 | 0.97 | 0.97 | 0.94 | 0.95 | 0.95 | 0.98 | 0.99 |  |  |  |
| Min | 0.77 | 0.83 | 0.80 | 0.69 | 0.83 | 0.83 | 0.94 | 0.94 |  |  |  |
|  |  |  |  |  |  |  |  |  |  |  |  |
|  | fold 1 | fold 2 | fold 3 | fold 4 | fold 5 | fold 6 | fold 7 | fold 8 | fold 9 | fold 10 | Median |
| Iterations | 28 | 21 | 27 | 18 | 22 | 38 | 15 | 38 | 15 | 27 | 24.5 |
| Intercepts | 0 | 0 | 0 | 0 | 0 | 0 | 0 | 0 | 0 | 0 | 0 |
| Features |  |  |  |  |  |  |  |  |  |  |  |
| Suicide-related CC | 2.38 | 2.74 | 2.54 | 2.62 | 2.18 | 2.38 | 2.40 | 2.48 | 2.41 | 2.80 | 2.44 |
| Patient Age | -0.11 | -0.05 | -0.08 | -0.05 | -0.09 | -0.07 | -0.10 | -0.09 | -0.07 | -0.10 | -0.08 |
| State ADI | -0.25 | -0.10 | -0.29 | -0.32 | -0.32 | -0.17 | -0.16 | -0.23 | -0.31 | -0.16 | -0.24 |
| National ADI | 0.02 | 0.00 | 0.03 | 0.03 | 0.03 | 0.01 | 0.01 | 0.01 | 0.03 | 0.01 | 0.02 |
| Site | -1.68 | -1.73 | -1.72 | -1.78 | -1.75 | -1.74 | -1.55 | -1.98 | -1.63 | -1.64 | -1.72 |
| Medical Admission | -0.62 | -0.41 | -0.52 | -0.35 | -0.62 | -0.21 | -0.62 | -0.86 | -0.53 | -0.40 | -0.52 |
| Psychiatric Admission | 0.67 | 0.81 | 0.90 | 0.80 | 0.80 | 0.64 | 0.65 | 0.85 | 0.71 | 0.60 | 0.75 |
| Involuntary Legal Status | 0.05 | 0.00 | 0.00 | 0.18 | 0.11 | 0.01 | 0.17 | 0.06 | 0.00 | 0.00 | 0.03 |
| Mental Health-related CC | 2.84 | 2.52 | 2.44 | 2.63 | 2.47 | 2.73 | 2.43 | 2.15 | 2.53 | 2.64 | 2.52 |
| Male | 0.00 | 0.25 | 0.11 | 0.11 | 0.12 | 0.01 | 0.00 | 0.00 | 0.05 | 0.08 | 0.06 |
| Non-Hispanic White | 0.00 | 0.00 | 0.00 | 0.00 | 0.00 | 0.00 | 0.00 | 0.00 | 0.00 | 0.14 | 0.00 |
| Non-Hispanic Black | 0.00 | 0.24 | 0.08 | 0.00 | 0.36 | 0.00 | 0.16 | 0.05 | 0.00 | 0.00 | 0.02 |
| Non-Hispanic Asian | 0.00 | 0.00 | -0.04 | 0.00 | 0.00 | 0.00 | -0.14 | 0.00 | -0.34 | 0.00 | 0.00 |
| Hispanic | 0.65 | 0.81 | 0.80 | 0.92 | 0.77 | 0.37 | 0.61 | 0.73 | 0.65 | 0.59 | 0.69 |
| Other Race/Ethnicity | -0.50 | -0.74 | -0.47 | -0.38 | -0.55 | -0.57 | -0.55 | -0.34 | -0.58 | -0.40 | -0.53 |
| ICD-10: Suicide or Self-Injury | 3.80 | 3.77 | 3.89 | 3.88 | 4.05 | 3.81 | 3.88 | 4.09 | 3.90 | 4.35 | 3.89 |
| prior_90d_ed | 0.00 | 0.00 | 0.00 | 0.00 | 0.00 | 0.00 | 0.00 | 0.00 | 0.00 | 0.00 | 0.00 |
| prior_180d_ed | 0.00 | 0.00 | 0.00 | 0.00 | 0.00 | 0.00 | 0.16 | 0.00 | 0.00 | 0.00 | 0.00 |
| prior_365d_ed | 0.38 | 0.30 | 0.31 | 0.31 | 0.31 | 0.33 | 0.12 | 0.28 | 0.29 | 0.15 | 0.30 |
| prior_90d_hosp | 0.00 | 0.00 | 0.00 | 0.00 | 0.00 | 0.00 | 0.00 | 0.00 | 0.00 | 0.00 | 0.00 |
| prior_180d_hosp | 0.00 | 0.00 | 0.00 | 0.00 | 0.00 | 0.00 | 0.00 | 0.00 | 0.00 | 0.00 | 0.00 |
| prior_365d_hosp | -1.31 | -1.26 | -0.96 | -1.20 | -0.96 | -0.91 | -1.05 | -0.94 | -0.99 | -1.05 | -1.02 |
| prior_90d_psych | 0.00 | 0.00 | 0.00 | 0.00 | 0.00 | 0.00 | 0.00 | 0.00 | 0.00 | 0.00 | 0.00 |
| prior_180d_psych | 0.00 | 0.00 | 0.00 | 0.00 | 0.00 | 0.00 | 0.00 | 0.00 | 0.00 | 0.00 | 0.00 |
| prior_365d_psych | -0.61 | 0.00 | -0.59 | -0.32 | -0.29 | -0.14 | -0.14 | -0.38 | 0.00 | 0.00 | -0.22 |
| acetaminophen_positive | 0.00 | 0.00 | 0.00 | 0.00 | 0.00 | 0.00 | 0.00 | 0.00 | 0.00 | 0.00 | 0.00 |
| salicylates_positive | 0.00 | 0.00 | 0.00 | 0.00 | 0.00 | 0.00 | 0.00 | 0.00 | 0.00 | 0.00 | 0.00 |
| benzodiazepines_positive | 0.00 | 0.00 | 0.00 | 0.00 | 0.00 | 0.00 | 0.00 | 0.00 | 0.00 | 0.00 | 0.00 |
| tricyclics_positive | 0.00 | 0.00 | 0.00 | 0.00 | 0.00 | 0.00 | 0.00 | 0.00 | 0.00 | 0.00 | 0.00 |
| alcohol_positive | 0.00 | 0.51 | 0.47 | 0.64 | 0.48 | 0.60 | 0.50 | 0.88 | 0.75 | 0.36 | 0.51 |
| any_od_lab_ordered_resulted | 0.00 | 0.00 | 0.00 | 0.00 | 0.00 | 0.00 | -0.11 | -0.26 | -0.22 | -0.14 | 0.00 |
| uds_amphetamine_methamphetamine_positive | 0.00 | 0.00 | 0.00 | 0.00 | 0.00 | 0.12 | 0.00 | 0.00 | 0.00 | 0.00 | 0.00 |
| uds_barbiturates_positive | 0.00 | 0.00 | 0.00 | 0.00 | 0.00 | 0.00 | 0.00 | 0.00 | 0.00 | 0.00 | 0.00 |
| uds_benzodiazepines_positive | 0.00 | 0.00 | 0.00 | 0.00 | 0.00 | 0.00 | 0.00 | 0.00 | 0.00 | 0.00 | 0.00 |
| uds_cannabinoids_positive | 0.00 | 0.00 | 0.00 | 0.00 | 0.00 | 0.00 | 0.00 | 0.00 | 0.00 | -0.05 | 0.00 |
| uds_cocaine_positive | 0.00 | 0.00 | 0.00 | 0.00 | 0.00 | 0.00 | 0.00 | 0.00 | 0.00 | 0.00 | 0.00 |
| uds_methadone_positive | 0.00 | 0.00 | 0.00 | 0.00 | 0.00 | 0.00 | 0.00 | 0.00 | 0.00 | 0.00 | 0.00 |
| uds_opiates_positive | 0.00 | 0.00 | 0.00 | 0.00 | 0.00 | 0.00 | 0.00 | 0.00 | 0.00 | 0.00 | 0.00 |
| uds_ethanol_positive | 0.00 | 0.00 | 0.00 | 0.00 | 0.00 | 0.00 | 0.00 | 0.00 | 0.00 | 0.00 | 0.00 |
| uds_oxycodone_positive | 0.00 | 0.00 | 0.00 | 0.00 | 0.00 | 0.00 | 0.00 | 0.00 | 0.00 | 0.00 | 0.00 |
| any_uds_ordered_resulted | 0.28 | 0.03 | 0.07 | 0.20 | 0.30 | 0.15 | 0.21 | 0.44 | 0.01 | 0.21 | 0.21 |
| encounter_year | 0.00 | 0.00 | 0.00 | 0.00 | 0.00 | 0.00 | 0.00 | 0.00 | 0.00 | 0.00 | 0.00 |
| provider_sex_male | 0.20 | 0.00 | 0.01 | 0.14 | 0.08 | 0.11 | 0.00 | 0.07 | 0.09 | 0.00 | 0.07 |
| Antidepressants | -0.27 | -0.10 | -0.21 | -0.18 | 0.00 | -0.11 | -0.10 | -0.07 | -0.01 | -0.13 | -0.11 |
| Antiepileptics | 0.00 | 0.00 | 0.00 | 0.00 | 0.00 | 0.00 | 0.00 | -0.04 | 0.00 | 0.00 | 0.00 |
| Antihistamines | -1.31 | -0.68 | -0.94 | -1.05 | -0.89 | -0.83 | -0.84 | -0.76 | -0.97 | -1.07 | -0.91 |
| Antipsychotics | 0.57 | 0.29 | 0.38 | 0.42 | 0.37 | 0.36 | 0.22 | 0.40 | 0.26 | 0.29 | 0.37 |
| Anxiolytics | 0.00 | 0.00 | 0.06 | 0.00 | 0.00 | 0.03 | 0.00 | 0.21 | 0.10 | 0.03 | 0.01 |
| Hypnotics | 0.00 | 0.00 | 0.00 | 0.00 | 0.00 | 0.00 | 0.00 | 0.00 | 0.00 | 0.00 | 0.00 |
| Lithium | 0.00 | 0.56 | 0.39 | 0.16 | 0.00 | 0.06 | 0.15 | 0.00 | 0.03 | 0.00 | 0.04 |
| Psychostimulants | 0.00 | -0.01 | -0.36 | 0.00 | 0.00 | 0.00 | 0.00 | 0.00 | 0.00 | 0.00 | 0.00 |
| Injectables | 1.11 | 0.65 | 0.64 | 0.63 | 0.27 | 0.62 | 0.85 | 0.26 | 0.71 | 0.83 | 0.64 |
| Number_of_med_classes | 0.00 | 0.00 | -0.04 | 0.00 | -0.14 | 0.00 | 0.00 | 0.00 | 0.00 | 0.00 | 0.00 |
|  |  |  |  |  |  |  |  |  |  |  |  |
|  |  |  |  |  |  |  |  |  |  |  |  |
| 3. Model 3: Lasso-penalized logistic regression |  |  |  |  |  |  |  |  |  |  |  |
|  | TP | FN | TN | FP |  |  |  |  |  |  |  |
| Count | 242 | 42 | 287 | 29 |  |  |  |  |  |  |  |
| Proportion of total N | 0.40 | 0.07 | 0.48 | 0.05 |  |  |  |  |  |  |  |
|  |  |  |  |  |  |  |  |  |  |  |  |
|  | Sensitivity | Specificity | PPV | NPV | Accuracy | F-Statistic (weighted avg) | ROC-AUC | PR-AUC |  |  |  |
| Mean | 0.86 | 0.91 | 0.89 | 0.87 | 0.88 | 0.88 | 0.97 | 0.97 |  |  |  |
| SD | 0.06 | 0.04 | 0.06 | 0.07 | 0.03 | 0.03 | 0.01 | 0.02 |  |  |  |
| 95%CI upper | 0.90 | 0.94 | 0.92 | 0.90 | 0.91 | 0.91 | 0.98 | 0.98 |  |  |  |
| 95%CI lower | 0.81 | 0.88 | 0.87 | 0.84 | 0.86 | 0.86 | 0.95 | 0.95 |  |  |  |
| Max | 0.96 | 0.97 | 0.97 | 0.97 | 0.97 | 0.97 | 0.99 | 0.99 |  |  |  |
| Min | 0.79 | 0.83 | 0.79 | 0.71 | 0.85 | 0.85 | 0.94 | 0.94 |  |  |  |
|  |  |  |  |  |  |  |  |  |  |  |  |
|  | fold 1 | fold 2 | fold 3 | fold 4 | fold 5 | fold 6 | fold 7 | fold 8 | fold 9 | fold 10 | Median |
| Iterations | 29 | 13 | 24 | 27 | 18 | 21 | 27 | 19 | 13 | 26 | 22.5 |
| Intercepts | 0 | 0 | 0 | 0 | 0 | 0 | 0 | 0 | 0 | 0 | 0 |
| Features |  |  |  |  |  |  |  |  |  |  |  |
| Suicide-related CC | 2.23 | 2.56 | 2.32 | 2.38 | 2.01 | 2.24 | 2.11 | 2.35 | 2.23 | 2.53 | 2.28 |
| Patient Age | -0.12 | -0.05 | -0.06 | -0.04 | -0.07 | -0.04 | -0.08 | -0.07 | -0.04 | -0.09 | -0.06 |
| State ADI | -0.21 | -0.10 | -0.27 | -0.30 | -0.34 | -0.19 | -0.19 | -0.22 | -0.26 | -0.11 | -0.22 |
| National ADI | 0.02 | 0.00 | 0.03 | 0.02 | 0.03 | 0.02 | 0.02 | 0.02 | 0.02 | 0.01 | 0.02 |
| Site | -1.53 | -1.60 | -1.58 | -1.66 | -1.36 | -1.57 | -1.38 | -1.80 | -1.57 | -1.45 | -1.57 |
| Medical Admission | -0.84 | -0.71 | -0.90 | -0.86 | -1.14 | -0.57 | -0.90 | -1.13 | -1.01 | -0.86 | -0.88 |
| Psychiatric Admission | 0.51 | 0.63 | 0.75 | 0.61 | 0.59 | 0.51 | 0.47 | 0.67 | 0.52 | 0.37 | 0.56 |
| Involuntary Legal Status | 0.00 | 0.00 | 0.00 | 0.00 | 0.00 | 0.00 | 0.03 | 0.00 | 0.00 | 0.00 | 0.00 |
| Mental Health-related CC | 2.58 | 2.28 | 2.22 | 2.44 | 2.25 | 2.51 | 2.23 | 1.95 | 2.42 | 2.44 | 2.35 |
| Male | 0.10 | 0.05 | 0.12 | 0.32 | 0.10 | 0.09 | 0.13 | 0.00 | 0.14 | 0.15 | 0.11 |
| Non-Hispanic White | 0.00 | 0.00 | 0.00 | 0.00 | 0.00 | 0.00 | 0.00 | 0.00 | 0.00 | 0.09 | 0.00 |
| Non-Hispanic Black | 0.04 | 0.47 | 0.19 | 0.00 | 0.54 | 0.00 | 0.42 | 0.17 | 0.00 | 0.00 | 0.10 |
| Non-Hispanic Asian | -0.06 | 0.00 | -0.13 | 0.00 | 0.00 | 0.00 | -0.15 | 0.00 | -0.37 | 0.00 | 0.00 |
| Hispanic | 0.60 | 0.90 | 0.90 | 1.11 | 0.87 | 0.43 | 0.83 | 0.86 | 0.70 | 0.53 | 0.84 |
| Other Race/Ethnicity | -0.42 | -0.62 | -0.42 | -0.26 | -0.42 | -0.57 | -0.52 | -0.34 | -0.57 | -0.45 | -0.44 |
| ICD 10 Group: Accidental or Undetermined Poisoning | 0.00 | 0.00 | 0.00 | 0.00 | 0.00 | 0.00 | 0.00 | 0.00 | 0.00 | 0.00 | 0.00 |
| ICD-10 Group: ADHD | 0.31 | 0.07 | 0.30 | 0.09 | 0.22 | 0.23 | 0.08 | 0.37 | 0.40 | 0.39 | 0.27 |
| ICD-10 Group: Anxiety Disorders | 0.00 | 0.29 | 0.00 | 0.23 | 0.22 | 0.17 | 0.32 | 0.25 | 0.00 | 0.27 | 0.22 |
| ICD-10 Group: Autism Spectrum Disorder | 0.09 | 0.00 | 0.00 | 0.05 | 0.28 | 0.23 | 0.00 | 0.10 | 0.00 | 0.03 | 0.04 |
| ICD-10 Group: Bipolar and Related Disorders | 0.00 | 0.00 | 0.00 | 0.27 | 0.01 | 0.00 | 0.09 | 0.00 | 0.35 | 0.00 | 0.00 |
| ICD-10 Group: Communication Disorders | 0.00 | 0.00 | 0.00 | 0.00 | 0.00 | 0.00 | 0.00 | 0.00 | 0.00 | 0.00 | 0.00 |
| ICD-10: Depressive Disorders | 0.91 | 0.97 | 0.82 | 0.88 | 0.86 | 0.74 | 0.91 | 0.51 | 0.54 | 0.99 | 0.87 |
| ICD-10 Group: Developmental Delay or Unspecified Neurodevelopmental Disorder | 0.00 | 0.00 | 0.07 | 0.00 | 0.00 | 0.00 | 0.00 | 0.00 | 0.00 | 0.00 | 0.00 |
| ICD-10 Group: Disruptive Impulse Control and Conduct Disorders | -0.23 | -0.35 | -0.22 | -0.37 | 0.00 | -0.11 | 0.00 | 0.00 | -0.32 | -0.41 | -0.22 |
| ICD-10 Group: Dissociative Disorders | 0.00 | 0.00 | 0.00 | 0.00 | 0.00 | 0.00 | 0.00 | 0.00 | 0.00 | 0.00 | 0.00 |
| ICD-10 Group: Elimination Disorders | 0.00 | 0.00 | 0.00 | 0.00 | 0.00 | 0.00 | 0.00 | 0.00 | 0.00 | 0.00 | 0.00 |
| ICD-10 Group: Feeding and Eating Disorders | 0.00 | 0.00 | 0.00 | 0.00 | 0.00 | 0.00 | 0.00 | 0.00 | 0.00 | 0.00 | 0.00 |
| ICD-10 Group: Fetal or Newborn Damage Related to Maternal Substance Abuse | 0.00 | 0.00 | 0.00 | 0.00 | 0.00 | 0.00 | 0.00 | 0.00 | 0.00 | 0.00 | 0.00 |
| ICD-10 Group: Intellectual Disability | 0.00 | 0.00 | 0.00 | 0.00 | 0.00 | 0.00 | 0.00 | 0.00 | 0.00 | 0.00 | 0.00 |
| ICD-10 Group: Maternal Mental Illness or Substance Abuse | 0.00 | 0.00 | 0.00 | 0.00 | 0.00 | 0.00 | 0.00 | 0.00 | 0.00 | 0.00 | 0.00 |
| ICD-10 Group: Mental Health Symptom | 0.35 | 0.54 | 0.49 | 0.45 | 0.63 | 0.43 | 0.44 | 0.61 | 0.33 | 0.56 | 0.47 |
| ICD-10 Group: Miscellaneous | 0.00 | 0.00 | 0.00 | 0.00 | 0.00 | 0.00 | 0.00 | -0.01 | 0.00 | 0.00 | 0.00 |
| ICD-10 Group: Motor Disorders | 0.00 | 0.00 | 0.00 | 0.00 | 0.00 | 0.00 | 0.00 | 0.00 | 0.00 | 0.00 | 0.00 |
| ICD-10 Group: Neurocognitive Disorders | 0.00 | 0.00 | 0.00 | 0.00 | 0.00 | 0.00 | 0.00 | 0.00 | 0.00 | 0.00 | 0.00 |
| ICD-10 Group: Obsessive Compulsive and Related Disorders | 0.00 | 0.00 | -0.08 | 0.00 | 0.00 | 0.00 | 0.00 | 0.00 | 0.00 | 0.00 | 0.00 |
| ICD-10 Group: Other Medical Condition | -0.13 | -0.16 | -0.17 | -0.09 | -0.12 | -0.13 | -0.15 | 0.00 | 0.00 | 0.00 | -0.12 |
| ICD-10 Group: Personality Disorders | 0.00 | 0.00 | 0.00 | 0.00 | 0.00 | 0.00 | 0.00 | 0.00 | 0.00 | 0.00 | 0.00 |
| ICD-10 Group: Schizophrenia Spectrum and Other Psychotic Disorders | -0.06 | 0.00 | -0.14 | 0.00 | -0.10 | -0.57 | -0.33 | -0.13 | -0.33 | -0.29 | -0.14 |
| ICD-10 Group: Sexuality and Gender Identity Disorders | 0.00 | 0.00 | 0.00 | 0.00 | 0.00 | 0.00 | 0.00 | 0.00 | 0.00 | 0.00 | 0.00 |
| ICD-10 Group: Sleep Wake Disorders | 0.00 | 0.00 | 0.00 | 0.00 | 0.00 | 0.00 | 0.00 | 0.00 | 0.00 | 0.00 | 0.00 |
| ICD-10 Group: Somatic Symptom and Related Disorders | 0.00 | 0.00 | 0.00 | 0.00 | 0.00 | 0.00 | 0.00 | 0.00 | 0.00 | 0.00 | 0.00 |
| ICD-10 Group: Specific Learning Disorders | 0.00 | 0.00 | 0.00 | 0.00 | 0.00 | 0.00 | 0.00 | 0.00 | 0.00 | 0.00 | 0.00 |
| ICD-10 Group: Substance Abuse Related Medical Illness | 0.00 | 0.00 | 0.00 | 0.00 | 0.00 | 0.00 | 0.00 | 0.00 | 0.00 | 0.00 | 0.00 |
| ICD-10 Group: Substance Related and Addictive Disorders | -0.06 | -0.06 | -0.14 | -0.26 | -0.49 | -0.06 | -0.16 | 0.00 | -0.15 | -0.16 | -0.14 |
| ICD-10: Suicide or Self-Injury | 3.75 | 3.62 | 3.79 | 3.84 | 4.12 | 3.75 | 3.86 | 4.09 | 3.89 | 4.29 | 3.85 |
| ICD-10 Group: Suicidal Ideation (Strict) | 0.00 | 0.00 | 0.00 | 0.00 | 0.00 | 0.00 | 0.00 | 0.00 | 0.00 | 0.00 | 0.00 |
| ICD-10 Group: Trauma- and Stressor-related Disorders | 0.82 | 0.84 | 1.16 | 1.20 | 1.44 | 0.98 | 0.60 | 0.87 | 1.71 | 0.95 | 0.97 |
| prior_90d_ed | 0.00 | 0.00 | 0.00 | 0.00 | 0.00 | 0.00 | 0.00 | 0.00 | 0.00 | 0.00 | 0.00 |
| prior_180d_ed | 0.00 | 0.00 | 0.00 | 0.00 | 0.00 | 0.00 | 0.14 | 0.00 | 0.00 | 0.00 | 0.00 |
| prior_365d_ed | 0.33 | 0.25 | 0.26 | 0.26 | 0.24 | 0.28 | 0.13 | 0.23 | 0.26 | 0.06 | 0.25 |
| prior_90d_hosp | 0.00 | 0.00 | 0.00 | 0.00 | 0.00 | 0.00 | 0.00 | 0.00 | 0.00 | 0.00 | 0.00 |
| prior_180d_hosp | 0.00 | 0.00 | 0.00 | 0.00 | 0.00 | 0.00 | 0.00 | 0.00 | 0.00 | 0.00 | 0.00 |
| prior_365d_hosp | -0.97 | -0.87 | -0.61 | -0.87 | -0.60 | -0.67 | -0.81 | -0.76 | -0.79 | -0.63 | -0.77 |
| prior_90d_psych | 0.00 | 0.00 | 0.00 | 0.00 | 0.00 | 0.00 | 0.00 | 0.00 | 0.00 | 0.00 | 0.00 |
| prior_180d_psych | 0.00 | 0.00 | 0.00 | 0.00 | 0.00 | 0.00 | 0.00 | 0.00 | 0.00 | 0.00 | 0.00 |
| prior_365d_psych | -0.30 | 0.00 | -0.17 | -0.06 | 0.00 | 0.00 | 0.00 | -0.06 | 0.00 | 0.00 | 0.00 |
| acetaminophen_positive | 0.00 | 0.00 | 0.00 | 0.00 | 0.00 | 0.00 | 0.00 | 0.00 | 0.00 | 0.00 | 0.00 |
| salicylates_positive | 0.00 | 0.00 | 0.00 | 0.00 | 0.00 | 0.00 | 0.00 | 0.00 | 0.00 | 0.00 | 0.00 |
| benzodiazepines_positive | 0.00 | 0.00 | 0.00 | 0.00 | 0.00 | 0.00 | 0.00 | 0.00 | 0.00 | 0.00 | 0.00 |
| tricyclics_positive | 0.00 | 0.00 | 0.00 | 0.00 | 0.00 | 0.00 | 0.00 | 0.00 | 0.00 | 0.00 | 0.00 |
| alcohol_positive | 0.00 | 0.70 | 0.63 | 0.73 | 0.26 | 0.58 | 0.32 | 0.77 | 0.57 | 0.32 | 0.57 |
| any_od_lab_ordered_resulted | 0.00 | 0.00 | 0.00 | 0.00 | 0.00 | 0.00 | -0.10 | -0.07 | -0.06 | -0.19 | 0.00 |
| uds_amphetamine_methamphetamine_positive | 0.00 | 0.00 | 0.00 | 0.00 | 0.00 | 0.18 | 0.00 | 0.00 | 0.00 | 0.00 | 0.00 |
| uds_barbiturates_positive | 0.00 | 0.00 | 0.00 | 0.00 | 0.00 | 0.00 | 0.00 | 0.00 | 0.00 | 0.00 | 0.00 |
| uds_benzodiazepines_positive | 0.00 | 0.00 | 0.00 | 0.00 | 0.00 | 0.00 | 0.00 | 0.00 | 0.00 | 0.00 | 0.00 |
| uds_cannabinoids_positive | 0.00 | 0.00 | 0.00 | 0.00 | 0.00 | 0.00 | 0.00 | 0.00 | 0.00 | 0.00 | 0.00 |
| uds_cocaine_positive | 0.00 | 0.00 | 0.00 | 0.00 | 0.00 | 0.00 | 0.00 | 0.00 | 0.00 | 0.00 | 0.00 |
| uds_methadone_positive | 0.00 | 0.00 | 0.00 | 0.00 | 0.00 | 0.00 | 0.00 | 0.00 | 0.00 | 0.00 | 0.00 |
| uds_opiates_positive | 0.00 | 0.00 | 0.00 | 0.00 | 0.00 | 0.00 | 0.00 | 0.00 | 0.00 | 0.00 | 0.00 |
| uds_ethanol_positive | 0.00 | 0.00 | 0.00 | 0.00 | 0.00 | 0.00 | 0.00 | 0.00 | 0.00 | 0.00 | 0.00 |
| uds_oxycodone_positive | 0.00 | 0.00 | 0.00 | 0.00 | 0.00 | 0.00 | 0.00 | 0.00 | 0.00 | 0.00 | 0.00 |
| any_uds_ordered_resulted | 0.28 | 0.04 | 0.05 | 0.24 | 0.37 | 0.26 | 0.29 | 0.36 | 0.05 | 0.35 | 0.27 |
| encounter_year | 0.00 | 0.00 | 0.00 | 0.00 | 0.00 | 0.00 | 0.00 | 0.00 | 0.00 | 0.00 | 0.00 |
| provider_sex_male | 0.23 | 0.38 | 0.10 | 0.15 | 0.20 | 0.12 | 0.07 | 0.13 | 0.16 | 0.17 | 0.16 |
| Antidepressants | -0.31 | -0.06 | -0.16 | -0.15 | 0.00 | -0.04 | -0.11 | -0.03 | 0.00 | -0.10 | -0.08 |
| Antiepileptics | 0.00 | 0.00 | 0.00 | 0.00 | 0.00 | 0.00 | 0.00 | 0.00 | 0.00 | 0.00 | 0.00 |
| Antihistamines | -1.38 | -0.53 | -0.87 | -0.95 | -0.72 | -0.82 | -0.61 | -0.66 | -0.80 | -0.99 | -0.81 |
| Antipsychotics | 0.33 | 0.23 | 0.26 | 0.47 | 0.21 | 0.24 | 0.21 | 0.34 | 0.24 | 0.25 | 0.25 |
| Anxiolytics | 0.00 | 0.00 | 0.00 | 0.00 | 0.00 | 0.05 | 0.00 | 0.02 | 0.08 | 0.01 | 0.00 |
| Hypnotics | 0.00 | 0.00 | 0.00 | 0.00 | 0.00 | 0.00 | 0.00 | 0.00 | 0.00 | 0.00 | 0.00 |
| Lithium | 0.00 | 0.87 | 0.51 | 0.35 | 0.00 | 0.34 | 0.41 | 0.48 | 0.25 | 0.18 | 0.34 |
| Psychostimulants | 0.00 | 0.00 | -0.05 | 0.00 | 0.00 | 0.00 | 0.00 | 0.00 | 0.00 | 0.00 | 0.00 |
| Injectables | 0.87 | 0.41 | 0.49 | 0.34 | 0.24 | 0.29 | 0.53 | 0.24 | 0.50 | 0.62 | 0.45 |
| Number_of_med_classes | 0.11 | 0.00 | 0.00 | 0.00 | -0.05 | 0.00 | 0.00 | 0.00 | 0.00 | 0.00 | 0.00 |
|  |  |  |  |  |  |  |  |  |  |  |  |
| 4. Model 1: Random Forest |  |  |  |  |  |  |  |  |  |  |  |
|  | TP | FN | TN | FP |  |  |  |  |  |  |  |
| Count | 241 | 43 | 288 | 28 |  |  |  |  |  |  |  |
| Proportion of total N | 0.40 | 0.07 | 0.48 | 0.05 |  |  |  |  |  |  |  |
|  |  |  |  |  |  |  |  |  |  |  |  |
|  | Sensitivity | Specificity | PPV | NPV | Accuracy | F-Statistic (weighted avg) | ROC-AUC | PR-AUC |  |  |  |
| Mean | 0.85 | 0.91 | 0.90 | 0.87 | 0.88 | 0.88 | 0.95 | 0.95 |  |  |  |
| SD | 0.05 | 0.04 | 0.05 | 0.07 | 0.03 | 0.03 | 0.03 | 0.02 |  |  |  |
| 95%CI upper | 0.90 | 0.94 | 0.92 | 0.89 | 0.91 | 0.91 | 0.97 | 0.97 |  |  |  |
| 95%CI lower | 0.81 | 0.88 | 0.87 | 0.84 | 0.86 | 0.86 | 0.93 | 0.94 |  |  |  |
| Max | 0.93 | 0.97 | 0.96 | 0.94 | 0.92 | 0.92 | 0.98 | 0.98 |  |  |  |
| Min | 0.79 | 0.86 | 0.82 | 0.70 | 0.83 | 0.84 | 0.90 | 0.92 |  |  |  |
|  |  |  |  |  |  |  |  |  |  |  |  |
|  | fold 1 | fold 2 | fold 3 | fold 4 | fold 5 | fold 6 | fold 7 | fold 8 | fold 9 | fold 10 | Median |
| Suicide-related CC | 0.10 | 0.10 | 0.10 | 0.13 | 0.11 | 0.10 | 0.11 | 0.11 | 0.10 | 0.09 | 0.10 |
| Mental Health-related CC | 0.19 | 0.18 | 0.17 | 0.18 | 0.16 | 0.18 | 0.18 | 0.16 | 0.18 | 0.17 | 0.18 |
| ICD 10 Group: Accidental or Undetermined Poisoning | 0.00 | 0.00 | 0.00 | 0.00 | 0.00 | 0.00 | 0.00 | 0.00 | 0.00 | 0.00 | 0.00 |
| ICD-10 Group: ADHD | 0.02 | 0.02 | 0.02 | 0.02 | 0.02 | 0.02 | 0.02 | 0.02 | 0.03 | 0.03 | 0.02 |
| ICD-10 Group: Anxiety Disorders | 0.02 | 0.03 | 0.03 | 0.02 | 0.02 | 0.02 | 0.02 | 0.02 | 0.02 | 0.02 | 0.02 |
| ICD-10 Group: Autism Spectrum Disorder | 0.02 | 0.02 | 0.02 | 0.02 | 0.02 | 0.02 | 0.02 | 0.02 | 0.02 | 0.02 | 0.02 |
| ICD-10 Group: Bipolar and Related Disorders | 0.01 | 0.01 | 0.01 | 0.01 | 0.01 | 0.01 | 0.01 | 0.01 | 0.01 | 0.01 | 0.01 |
| ICD-10 Group: Communication Disorders | 0.00 | 0.00 | 0.00 | 0.00 | 0.00 | 0.00 | 0.00 | 0.00 | 0.00 | 0.00 | 0.00 |
| ICD-10: Depressive Disorders | 0.08 | 0.07 | 0.06 | 0.06 | 0.07 | 0.07 | 0.07 | 0.06 | 0.07 | 0.07 | 0.07 |
| ICD-10 Group: Developmental Delay or Unspecified Neurodevelopmental Disorder | 0.01 | 0.00 | 0.00 | 0.00 | 0.01 | 0.00 | 0.00 | 0.01 | 0.01 | 0.01 | 0.01 |
| ICD-10 Group: Disruptive Impulse Control and Conduct Disorders | 0.01 | 0.02 | 0.01 | 0.01 | 0.02 | 0.02 | 0.02 | 0.02 | 0.01 | 0.02 | 0.02 |
| ICD-10 Group: Dissociative Disorders | 0.00 | 0.00 | 0.00 | 0.00 | 0.00 | 0.00 | 0.00 | 0.00 | 0.00 | 0.00 | 0.00 |
| ICD-10 Group: Elimination Disorders | 0.00 | 0.00 | 0.00 | 0.00 | 0.00 | 0.00 | 0.00 | 0.00 | 0.00 | 0.00 | 0.00 |
| ICD-10 Group: Feeding and Eating Disorders | 0.01 | 0.00 | 0.01 | 0.01 | 0.01 | 0.01 | 0.01 | 0.01 | 0.01 | 0.01 | 0.01 |
| ICD-10 Group: Fetal or Newborn Damage Related to Maternal Substance Abuse | 0.00 | 0.00 | 0.00 | 0.00 | 0.00 | 0.00 | 0.00 | 0.00 | 0.00 | 0.00 | 0.00 |
| ICD-10 Group: Intellectual Disability | 0.00 | 0.00 | 0.00 | 0.00 | 0.00 | 0.00 | 0.00 | 0.00 | 0.00 | 0.00 | 0.00 |
| ICD-10 Group: Maternal Mental Illness or Substance Abuse | 0.00 | 0.00 | 0.00 | 0.00 | 0.00 | 0.00 | 0.00 | 0.00 | 0.00 | 0.00 | 0.00 |
| ICD-10 Group: Mental Health Symptom | 0.03 | 0.03 | 0.03 | 0.03 | 0.04 | 0.03 | 0.03 | 0.04 | 0.03 | 0.03 | 0.03 |
| ICD-10 Group: Miscellaneous | 0.01 | 0.02 | 0.02 | 0.02 | 0.02 | 0.02 | 0.02 | 0.02 | 0.01 | 0.02 | 0.02 |
| ICD-10 Group: Motor Disorders | 0.00 | 0.00 | 0.00 | 0.00 | 0.00 | 0.00 | 0.00 | 0.00 | 0.00 | 0.00 | 0.00 |
| ICD-10 Group: Neurocognitive Disorders | 0.00 | 0.00 | 0.00 | 0.00 | 0.00 | 0.00 | 0.00 | 0.00 | 0.00 | 0.00 | 0.00 |
| ICD-10 Group: Obsessive Compulsive and Related Disorders | 0.02 | 0.01 | 0.01 | 0.01 | 0.01 | 0.01 | 0.01 | 0.01 | 0.01 | 0.01 | 0.01 |
| ICD-10 Group: Other Medical Condition | 0.05 | 0.05 | 0.04 | 0.04 | 0.04 | 0.04 | 0.04 | 0.04 | 0.04 | 0.04 | 0.04 |
| ICD-10 Group: Personality Disorders | 0.01 | 0.01 | 0.01 | 0.01 | 0.00 | 0.00 | 0.01 | 0.00 | 0.01 | 0.00 | 0.01 |
| ICD-10 Group: Schizophrenia Spectrum and Other Psychotic Disorders | 0.00 | 0.01 | 0.01 | 0.01 | 0.01 | 0.01 | 0.01 | 0.01 | 0.01 | 0.01 | 0.01 |
| ICD-10 Group: Sexuality and Gender Identity Disorders | 0.00 | 0.00 | 0.00 | 0.00 | 0.00 | 0.00 | 0.00 | 0.00 | 0.00 | 0.00 | 0.00 |
| ICD-10 Group: Sleep Wake Disorders | 0.00 | 0.00 | 0.00 | 0.00 | 0.00 | 0.00 | 0.00 | 0.00 | 0.00 | 0.00 | 0.00 |
| ICD-10 Group: Somatic Symptom and Related Disorders | 0.00 | 0.00 | 0.00 | 0.00 | 0.00 | 0.00 | 0.00 | 0.00 | 0.00 | 0.00 | 0.00 |
| ICD-10 Group: Specific Learning Disorders | 0.00 | 0.00 | 0.00 | 0.00 | 0.00 | 0.00 | 0.00 | 0.00 | 0.00 | 0.00 | 0.00 |
| ICD-10 Group: Substance Abuse Related Medical Illness | 0.00 | 0.00 | 0.00 | 0.00 | 0.00 | 0.00 | 0.00 | 0.00 | 0.00 | 0.00 | 0.00 |
| ICD-10 Group: Substance Related and Addictive Disorders | 0.02 | 0.02 | 0.02 | 0.02 | 0.02 | 0.02 | 0.02 | 0.02 | 0.02 | 0.02 | 0.02 |
| ICD-10: Suicide or Self-Injury | 0.22 | 0.27 | 0.25 | 0.22 | 0.19 | 0.22 | 0.24 | 0.25 | 0.24 | 0.23 | 0.23 |
| ICD-10 Group: Suicidal Ideation (Strict) | 0.15 | 0.12 | 0.16 | 0.16 | 0.21 | 0.17 | 0.15 | 0.14 | 0.14 | 0.18 | 0.16 |
| ICD-10 Group: Trauma- and Stressor-related Disorders | 0.01 | 0.01 | 0.01 | 0.01 | 0.01 | 0.01 | 0.01 | 0.01 | 0.01 | 0.01 | 0.01 |
|  |  |  |  |  |  |  |  |  |  |  |  |
|  |  |  |  |  |  |  |  |  |  |  |  |
| 2. Model 2: Random Forest |  |  |  |  |  |  |  |  |  |  |  |
|  | TP | FN | TN | FP |  |  |  |  |  |  |  |
| Count | 242 | 42 | 277 | 39 |  |  |  |  |  |  |  |
| Proportion of total N | 0.40 | 0.07 | 0.46 | 0.07 |  |  |  |  |  |  |  |
|  |  |  |  |  |  |  |  |  |  |  |  |
|  | Sensitivity | Specificity | PPV | NPV | Accuracy | F-Statistic (weighted avg) | ROC-AUC | PR-AUC |  |  |  |
| Mean | 0.85 | 0.88 | 0.86 | 0.87 | 0.87 | 0.87 | 0.97 | 0.96 |  |  |  |
| SD | 0.05 | 0.07 | 0.09 | 0.06 | 0.03 | 0.03 | 0.01 | 0.02 |  |  |  |
| 95%CI upper | 0.90 | 0.92 | 0.89 | 0.89 | 0.89 | 0.89 | 0.98 | 0.98 |  |  |  |
| 95%CI lower | 0.81 | 0.85 | 0.83 | 0.84 | 0.84 | 0.84 | 0.95 | 0.95 |  |  |  |
| Max | 0.93 | 1.00 | 1.00 | 0.94 | 0.92 | 0.92 | 0.98 | 0.99 |  |  |  |
| Min | 0.79 | 0.80 | 0.73 | 0.72 | 0.82 | 0.82 | 0.94 | 0.93 |  |  |  |
|  |  |  |  |  |  |  |  |  |  |  |  |
|  | fold 1 | fold 2 | fold 3 | fold 4 | fold 5 | fold 6 | fold 7 | fold 8 | fold 9 | fold 10 | Median |
| Suicide-related CC | 0.11 | 0.09 | 0.12 | 0.12 | 0.09 | 0.10 | 0.11 | 0.11 | 0.10 | 0.10 | 0.11 |
| Patient Age | 0.05 | 0.05 | 0.04 | 0.04 | 0.05 | 0.04 | 0.05 | 0.05 | 0.05 | 0.05 | 0.05 |
| State ADI | 0.04 | 0.03 | 0.04 | 0.03 | 0.03 | 0.03 | 0.03 | 0.03 | 0.04 | 0.04 | 0.03 |
| National ADI | 0.05 | 0.04 | 0.05 | 0.05 | 0.05 | 0.05 | 0.05 | 0.05 | 0.05 | 0.05 | 0.05 |
| Site | 0.04 | 0.04 | 0.04 | 0.04 | 0.04 | 0.04 | 0.04 | 0.04 | 0.04 | 0.04 | 0.04 |
| Medical Admission | 0.02 | 0.02 | 0.02 | 0.02 | 0.02 | 0.02 | 0.02 | 0.02 | 0.02 | 0.02 | 0.02 |
| Psychiatric Admission | 0.07 | 0.08 | 0.07 | 0.07 | 0.06 | 0.06 | 0.06 | 0.07 | 0.07 | 0.07 | 0.07 |
| Involuntary Legal Status | 0.02 | 0.03 | 0.02 | 0.03 | 0.03 | 0.02 | 0.03 | 0.03 | 0.02 | 0.03 | 0.03 |
| Mental Health-related CC | 0.16 | 0.16 | 0.13 | 0.15 | 0.16 | 0.16 | 0.15 | 0.13 | 0.16 | 0.15 | 0.15 |
| Male | 0.01 | 0.01 | 0.01 | 0.01 | 0.01 | 0.01 | 0.01 | 0.01 | 0.01 | 0.01 | 0.01 |
| Non-Hispanic White | 0.01 | 0.01 | 0.01 | 0.01 | 0.01 | 0.01 | 0.01 | 0.01 | 0.01 | 0.01 | 0.01 |
| Non-Hispanic Black | 0.01 | 0.01 | 0.01 | 0.01 | 0.01 | 0.01 | 0.00 | 0.01 | 0.01 | 0.01 | 0.01 |
| Non-Hispanic Asian | 0.00 | 0.00 | 0.00 | 0.00 | 0.00 | 0.00 | 0.00 | 0.00 | 0.00 | 0.00 | 0.00 |
| Hispanic | 0.01 | 0.01 | 0.01 | 0.01 | 0.01 | 0.01 | 0.01 | 0.01 | 0.01 | 0.01 | 0.01 |
| Other Race/Ethnicity | 0.01 | 0.01 | 0.01 | 0.01 | 0.01 | 0.01 | 0.01 | 0.01 | 0.01 | 0.01 | 0.01 |
| ICD-10: Suicide or Self-Injury | 0.20 | 0.22 | 0.23 | 0.19 | 0.23 | 0.24 | 0.23 | 0.24 | 0.21 | 0.21 | 0.22 |
| prior_90d_ed | 0.01 | 0.01 | 0.01 | 0.01 | 0.01 | 0.00 | 0.00 | 0.01 | 0.01 | 0.00 | 0.01 |
| prior_180d_ed | 0.01 | 0.01 | 0.01 | 0.01 | 0.01 | 0.01 | 0.01 | 0.01 | 0.01 | 0.01 | 0.01 |
| prior_365d_ed | 0.01 | 0.01 | 0.01 | 0.01 | 0.01 | 0.01 | 0.01 | 0.01 | 0.01 | 0.01 | 0.01 |
| prior_90d_hosp | 0.00 | 0.00 | 0.00 | 0.00 | 0.00 | 0.00 | 0.00 | 0.00 | 0.00 | 0.00 | 0.00 |
| prior_180d_hosp | 0.00 | 0.00 | 0.00 | 0.00 | 0.00 | 0.00 | 0.00 | 0.00 | 0.00 | 0.00 | 0.00 |
| prior_365d_hosp | 0.00 | 0.00 | 0.00 | 0.00 | 0.00 | 0.00 | 0.00 | 0.01 | 0.00 | 0.00 | 0.00 |
| prior_90d_psych | 0.00 | 0.00 | 0.00 | 0.00 | 0.00 | 0.00 | 0.00 | 0.00 | 0.00 | 0.00 | 0.00 |
| prior_180d_psych | 0.00 | 0.00 | 0.00 | 0.00 | 0.00 | 0.00 | 0.00 | 0.00 | 0.00 | 0.00 | 0.00 |
| prior_365d_psych | 0.00 | 0.00 | 0.00 | 0.00 | 0.00 | 0.00 | 0.00 | 0.00 | 0.00 | 0.00 | 0.00 |
| acetaminophen_positive | 0.00 | 0.00 | 0.00 | 0.00 | 0.00 | 0.00 | 0.00 | 0.00 | 0.00 | 0.00 | 0.00 |
| salicylates_positive | 0.00 | 0.00 | 0.00 | 0.00 | 0.00 | 0.00 | 0.00 | 0.00 | 0.00 | 0.00 | 0.00 |
| benzodiazepines_positive | 0.00 | 0.00 | 0.00 | 0.00 | 0.00 | 0.00 | 0.00 | 0.00 | 0.00 | 0.00 | 0.00 |
| tricyclics_positive | 0.00 | 0.00 | 0.00 | 0.00 | 0.00 | 0.00 | 0.00 | 0.00 | 0.00 | 0.00 | 0.00 |
| alcohol_positive | 0.00 | 0.00 | 0.00 | 0.01 | 0.00 | 0.01 | 0.00 | 0.00 | 0.01 | 0.01 | 0.00 |
| any_od_lab_ordered_resulted | 0.01 | 0.01 | 0.01 | 0.01 | 0.01 | 0.01 | 0.01 | 0.01 | 0.01 | 0.01 | 0.01 |
| uds_amphetamine_methamphetamine_positive | 0.00 | 0.00 | 0.00 | 0.00 | 0.00 | 0.00 | 0.00 | 0.00 | 0.00 | 0.00 | 0.00 |
| uds_barbiturates_positive | 0.00 | 0.00 | 0.00 | 0.00 | 0.00 | 0.00 | 0.00 | 0.00 | 0.00 | 0.00 | 0.00 |
| uds_benzodiazepines_positive | 0.00 | 0.00 | 0.00 | 0.00 | 0.00 | 0.00 | 0.00 | 0.00 | 0.00 | 0.00 | 0.00 |
| uds_cannabinoids_positive | 0.00 | 0.00 | 0.01 | 0.00 | 0.01 | 0.00 | 0.00 | 0.00 | 0.00 | 0.00 | 0.00 |
| uds_cocaine_positive | 0.00 | 0.00 | 0.00 | 0.00 | 0.00 | 0.00 | 0.00 | 0.00 | 0.00 | 0.00 | 0.00 |
| uds_methadone_positive | 0.00 | 0.00 | 0.00 | 0.00 | 0.00 | 0.00 | 0.00 | 0.00 | 0.00 | 0.00 | 0.00 |
| uds_opiates_positive | 0.00 | 0.00 | 0.00 | 0.00 | 0.00 | 0.00 | 0.00 | 0.00 | 0.00 | 0.00 | 0.00 |
| uds_ethanol_positive | 0.00 | 0.00 | 0.00 | 0.00 | 0.00 | 0.00 | 0.00 | 0.00 | 0.00 | 0.00 | 0.00 |
| uds_oxycodone_positive | 0.00 | 0.00 | 0.00 | 0.00 | 0.00 | 0.00 | 0.00 | 0.00 | 0.00 | 0.00 | 0.00 |
| any_uds_ordered_resulted | 0.03 | 0.03 | 0.02 | 0.03 | 0.04 | 0.03 | 0.03 | 0.03 | 0.03 | 0.04 | 0.03 |
| encounter_year | 0.02 | 0.02 | 0.03 | 0.02 | 0.02 | 0.03 | 0.03 | 0.02 | 0.03 | 0.03 | 0.03 |
| provider_sex_male | 0.01 | 0.01 | 0.01 | 0.01 | 0.01 | 0.01 | 0.01 | 0.01 | 0.01 | 0.01 | 0.01 |
| Antidepressants | 0.01 | 0.01 | 0.01 | 0.01 | 0.01 | 0.01 | 0.01 | 0.01 | 0.01 | 0.01 | 0.01 |
| Antiepileptics | 0.00 | 0.00 | 0.00 | 0.00 | 0.00 | 0.00 | 0.00 | 0.00 | 0.00 | 0.00 | 0.00 |
| Antihistamines | 0.00 | 0.00 | 0.00 | 0.01 | 0.00 | 0.00 | 0.00 | 0.01 | 0.00 | 0.00 | 0.00 |
| Antipsychotics | 0.01 | 0.01 | 0.01 | 0.01 | 0.01 | 0.01 | 0.01 | 0.01 | 0.01 | 0.01 | 0.01 |
| Anxiolytics | 0.01 | 0.01 | 0.01 | 0.01 | 0.01 | 0.01 | 0.01 | 0.01 | 0.01 | 0.01 | 0.01 |
| Hypnotics | 0.00 | 0.00 | 0.00 | 0.00 | 0.00 | 0.00 | 0.00 | 0.00 | 0.00 | 0.00 | 0.00 |
| Lithium | 0.00 | 0.00 | 0.00 | 0.00 | 0.00 | 0.00 | 0.00 | 0.00 | 0.00 | 0.00 | 0.00 |
| Psychostimulants | 0.01 | 0.01 | 0.01 | 0.01 | 0.01 | 0.01 | 0.01 | 0.00 | 0.00 | 0.01 | 0.01 |
| Injectables | 0.00 | 0.00 | 0.00 | 0.00 | 0.00 | 0.00 | 0.00 | 0.00 | 0.01 | 0.00 | 0.00 |
| Number_of_med_classes | 0.02 | 0.02 | 0.02 | 0.02 | 0.01 | 0.02 | 0.02 | 0.01 | 0.02 | 0.01 | 0.02 |
|  |  |  |  |  |  |  |  |  |  |  |  |
| 3. Model 3: Random Forest |  |  |  |  |  |  |  |  |  |  |  |
| Count | 243 | 41 | 290 | 26 |  |  |  |  |  |  |  |
| Proportion of total N | 0.41 | 0.07 | 0.48 | 0.04 |  |  |  |  |  |  |  |
|  |  |  |  |  |  |  |  |  |  |  |  |
|  | Sensitivity | Specificity | PPV | NPV | Accuracy | F-Statistic (weighted avg) | ROC-AUC | PR-AUC |  |  |  |
| Mean | 0.86 | 0.92 | 0.90 | 0.87 | 0.89 | 0.89 | 0.96 | 0.96 |  |  |  |
| SD | 0.06 | 0.05 | 0.07 | 0.07 | 0.04 | 0.04 | 0.02 | 0.03 |  |  |  |
| 95%CI upper | 0.90 | 0.95 | 0.93 | 0.90 | 0.91 | 0.91 | 0.98 | 0.98 |  |  |  |
| 95%CI lower | 0.81 | 0.89 | 0.88 | 0.85 | 0.86 | 0.86 | 0.95 | 0.95 |  |  |  |
| Max | 0.96 | 0.97 | 0.97 | 0.97 | 0.95 | 0.95 | 0.98 | 0.98 |  |  |  |
| Min | 0.76 | 0.83 | 0.78 | 0.71 | 0.83 | 0.83 | 0.93 | 0.90 |  |  |  |
|  |  |  |  |  |  |  |  |  |  |  |  |
|  | fold 1 | fold 2 | fold 3 | fold 4 | fold 5 | fold 6 | fold 7 | fold 8 | fold 9 | fold 10 | Median |
| Suicide-related CC | 0.05 | 0.07 | 0.08 | 0.08 | 0.08 | 0.08 | 0.08 | 0.07 | 0.06 | 0.09 | 0.08 |
| Patient Age | 0.03 | 0.03 | 0.03 | 0.03 | 0.03 | 0.03 | 0.03 | 0.03 | 0.03 | 0.03 | 0.03 |
| State ADI | 0.02 | 0.02 | 0.03 | 0.03 | 0.02 | 0.03 | 0.03 | 0.03 | 0.03 | 0.02 | 0.03 |
| National ADI | 0.03 | 0.03 | 0.03 | 0.03 | 0.03 | 0.03 | 0.03 | 0.03 | 0.03 | 0.03 | 0.03 |
| Site | 0.03 | 0.03 | 0.03 | 0.03 | 0.02 | 0.03 | 0.03 | 0.04 | 0.03 | 0.03 | 0.03 |
| Medical Admission | 0.02 | 0.01 | 0.02 | 0.02 | 0.01 | 0.01 | 0.01 | 0.01 | 0.01 | 0.02 | 0.01 |
| Psychiatric Admission | 0.05 | 0.06 | 0.05 | 0.03 | 0.05 | 0.06 | 0.05 | 0.04 | 0.05 | 0.05 | 0.05 |
| Involuntary Legal Status | 0.01 | 0.02 | 0.01 | 0.02 | 0.02 | 0.02 | 0.02 | 0.01 | 0.02 | 0.01 | 0.02 |
| Mental Health-related CC | 0.13 | 0.13 | 0.12 | 0.13 | 0.12 | 0.13 | 0.12 | 0.14 | 0.12 | 0.13 | 0.13 |
| Male | 0.01 | 0.01 | 0.01 | 0.01 | 0.01 | 0.01 | 0.01 | 0.01 | 0.01 | 0.01 | 0.01 |
| Non-Hispanic White | 0.01 | 0.01 | 0.01 | 0.01 | 0.01 | 0.01 | 0.01 | 0.01 | 0.01 | 0.01 | 0.01 |
| Non-Hispanic Black | 0.01 | 0.00 | 0.01 | 0.00 | 0.01 | 0.00 | 0.00 | 0.00 | 0.00 | 0.00 | 0.00 |
| Non-Hispanic Asian | 0.00 | 0.00 | 0.00 | 0.00 | 0.00 | 0.00 | 0.00 | 0.00 | 0.00 | 0.00 | 0.00 |
| Hispanic | 0.01 | 0.01 | 0.01 | 0.01 | 0.01 | 0.01 | 0.01 | 0.01 | 0.01 | 0.01 | 0.01 |
| Other Race/Ethnicity | 0.01 | 0.01 | 0.00 | 0.01 | 0.01 | 0.01 | 0.01 | 0.01 | 0.00 | 0.00 | 0.01 |
| ICD 10 Group: Accidental or Undetermined Poisoning | 0.00 | 0.00 | 0.00 | 0.00 | 0.00 | 0.00 | 0.00 | 0.00 | 0.00 | 0.00 | 0.00 |
| ICD-10 Group: ADHD | 0.01 | 0.01 | 0.01 | 0.01 | 0.01 | 0.01 | 0.01 | 0.01 | 0.01 | 0.01 | 0.01 |
| ICD-10 Group: Anxiety Disorders | 0.01 | 0.01 | 0.01 | 0.01 | 0.01 | 0.01 | 0.01 | 0.01 | 0.01 | 0.01 | 0.01 |
| ICD-10 Group: Autism Spectrum Disorder | 0.01 | 0.01 | 0.01 | 0.00 | 0.01 | 0.00 | 0.00 | 0.01 | 0.01 | 0.01 | 0.01 |
| ICD-10 Group: Bipolar and Related Disorders | 0.00 | 0.00 | 0.01 | 0.00 | 0.00 | 0.00 | 0.01 | 0.00 | 0.00 | 0.00 | 0.00 |
| ICD-10 Group: Communication Disorders | 0.00 | 0.00 | 0.00 | 0.00 | 0.00 | 0.00 | 0.00 | 0.00 | 0.00 | 0.00 | 0.00 |
| ICD-10: Depressive Disorders | 0.04 | 0.05 | 0.05 | 0.05 | 0.05 | 0.05 | 0.05 | 0.04 | 0.04 | 0.04 | 0.05 |
| ICD-10 Group: Developmental Delay or Unspecified Neurodevelopmental Disorder | 0.00 | 0.00 | 0.00 | 0.00 | 0.00 | 0.00 | 0.00 | 0.00 | 0.00 | 0.00 | 0.00 |
| ICD-10 Group: Disruptive Impulse Control and Conduct Disorders | 0.01 | 0.01 | 0.01 | 0.01 | 0.01 | 0.01 | 0.01 | 0.01 | 0.01 | 0.01 | 0.01 |
| ICD-10 Group: Dissociative Disorders | 0.00 | 0.00 | 0.00 | 0.00 | 0.00 | 0.00 | 0.00 | 0.00 | 0.00 | 0.00 | 0.00 |
| ICD-10 Group: Elimination Disorders | 0.00 | 0.00 | 0.00 | 0.00 | 0.00 | 0.00 | 0.00 | 0.00 | 0.00 | 0.00 | 0.00 |
| ICD-10 Group: Feeding and Eating Disorders | 0.00 | 0.00 | 0.00 | 0.00 | 0.00 | 0.00 | 0.00 | 0.00 | 0.00 | 0.00 | 0.00 |
| ICD-10 Group: Fetal or Newborn Damage Related to Maternal Substance Abuse | 0.00 | 0.00 | 0.00 | 0.00 | 0.00 | 0.00 | 0.00 | 0.00 | 0.00 | 0.00 | 0.00 |
| ICD-10 Group: Intellectual Disability | 0.00 | 0.00 | 0.00 | 0.00 | 0.00 | 0.00 | 0.00 | 0.00 | 0.00 | 0.00 | 0.00 |
| ICD-10 Group: Maternal Mental Illness or Substance Abuse | 0.00 | 0.00 | 0.00 | 0.00 | 0.00 | 0.00 | 0.00 | 0.00 | 0.00 | 0.00 | 0.00 |
| ICD-10 Group: Mental Health Symptom | 0.01 | 0.01 | 0.01 | 0.01 | 0.01 | 0.01 | 0.01 | 0.01 | 0.01 | 0.02 | 0.01 |
| ICD-10 Group: Miscellaneous | 0.00 | 0.01 | 0.01 | 0.01 | 0.01 | 0.01 | 0.01 | 0.01 | 0.01 | 0.01 | 0.01 |
| ICD-10 Group: Motor Disorders | 0.00 | 0.00 | 0.00 | 0.00 | 0.00 | 0.00 | 0.00 | 0.00 | 0.00 | 0.00 | 0.00 |
| ICD-10 Group: Neurocognitive Disorders | 0.00 | 0.00 | 0.00 | 0.00 | 0.00 | 0.00 | 0.00 | 0.00 | 0.00 | 0.00 | 0.00 |
| ICD-10 Group: Obsessive Compulsive and Related Disorders | 0.00 | 0.00 | 0.00 | 0.00 | 0.00 | 0.00 | 0.00 | 0.00 | 0.00 | 0.00 | 0.00 |
| ICD-10 Group: Other Medical Condition | 0.02 | 0.02 | 0.02 | 0.02 | 0.02 | 0.02 | 0.02 | 0.02 | 0.02 | 0.02 | 0.02 |
| ICD-10 Group: Personality Disorders | 0.00 | 0.00 | 0.00 | 0.00 | 0.00 | 0.00 | 0.00 | 0.00 | 0.00 | 0.00 | 0.00 |
| ICD-10 Group: Schizophrenia Spectrum and Other Psychotic Disorders | 0.00 | 0.00 | 0.00 | 0.00 | 0.00 | 0.00 | 0.00 | 0.00 | 0.00 | 0.00 | 0.00 |
| ICD-10 Group: Sexuality and Gender Identity Disorders | 0.00 | 0.00 | 0.00 | 0.00 | 0.00 | 0.00 | 0.00 | 0.00 | 0.00 | 0.00 | 0.00 |
| ICD-10 Group: Sleep Wake Disorders | 0.00 | 0.00 | 0.00 | 0.00 | 0.00 | 0.00 | 0.00 | 0.00 | 0.00 | 0.00 | 0.00 |
| ICD-10 Group: Somatic Symptom and Related Disorders | 0.00 | 0.00 | 0.00 | 0.00 | 0.00 | 0.00 | 0.00 | 0.00 | 0.00 | 0.00 | 0.00 |
| ICD-10 Group: Specific Learning Disorders | 0.00 | 0.00 | 0.00 | 0.00 | 0.00 | 0.00 | 0.00 | 0.00 | 0.00 | 0.00 | 0.00 |
| ICD-10 Group: Substance Abuse Related Medical Illness | 0.00 | 0.00 | 0.00 | 0.00 | 0.00 | 0.00 | 0.00 | 0.00 | 0.00 | 0.00 | 0.00 |
| ICD-10 Group: Substance Related and Addictive Disorders | 0.01 | 0.01 | 0.01 | 0.01 | 0.01 | 0.01 | 0.01 | 0.01 | 0.01 | 0.01 | 0.01 |
| ICD-10: Suicide or Self-Injury | 0.15 | 0.17 | 0.15 | 0.16 | 0.17 | 0.14 | 0.16 | 0.12 | 0.17 | 0.17 | 0.16 |
| ICD-10 Group: Suicidal Ideation (Strict) | 0.13 | 0.10 | 0.12 | 0.11 | 0.13 | 0.12 | 0.13 | 0.16 | 0.14 | 0.11 | 0.13 |
| ICD-10 Group: Trauma- and Stressor-related Disorders | 0.00 | 0.00 | 0.00 | 0.01 | 0.01 | 0.00 | 0.00 | 0.00 | 0.00 | 0.00 | 0.00 |
| prior_90d_ed | 0.01 | 0.01 | 0.00 | 0.00 | 0.01 | 0.01 | 0.00 | 0.00 | 0.00 | 0.00 | 0.00 |
| prior_180d_ed | 0.01 | 0.01 | 0.01 | 0.01 | 0.01 | 0.01 | 0.00 | 0.01 | 0.01 | 0.01 | 0.01 |
| prior_365d_ed | 0.01 | 0.01 | 0.01 | 0.01 | 0.01 | 0.01 | 0.01 | 0.01 | 0.01 | 0.01 | 0.01 |
| prior_90d_hosp | 0.00 | 0.00 | 0.00 | 0.00 | 0.00 | 0.00 | 0.00 | 0.00 | 0.00 | 0.00 | 0.00 |
| prior_180d_hosp | 0.01 | 0.00 | 0.00 | 0.00 | 0.00 | 0.00 | 0.00 | 0.00 | 0.00 | 0.00 | 0.00 |
| prior_365d_hosp | 0.00 | 0.00 | 0.00 | 0.00 | 0.00 | 0.00 | 0.00 | 0.00 | 0.00 | 0.00 | 0.00 |
| prior_90d_psych | 0.00 | 0.00 | 0.00 | 0.00 | 0.00 | 0.00 | 0.00 | 0.00 | 0.00 | 0.00 | 0.00 |
| prior_180d_psych | 0.00 | 0.00 | 0.00 | 0.00 | 0.00 | 0.00 | 0.00 | 0.00 | 0.00 | 0.00 | 0.00 |
| prior_365d_psych | 0.00 | 0.00 | 0.00 | 0.00 | 0.00 | 0.00 | 0.00 | 0.00 | 0.00 | 0.00 | 0.00 |
| acetaminophen_positive | 0.00 | 0.00 | 0.00 | 0.00 | 0.00 | 0.00 | 0.00 | 0.00 | 0.00 | 0.00 | 0.00 |
| salicylates_positive | 0.00 | 0.00 | 0.00 | 0.00 | 0.00 | 0.00 | 0.00 | 0.00 | 0.00 | 0.00 | 0.00 |
| benzodiazepines_positive | 0.00 | 0.00 | 0.00 | 0.00 | 0.00 | 0.00 | 0.00 | 0.00 | 0.00 | 0.00 | 0.00 |
| tricyclics_positive | 0.00 | 0.00 | 0.00 | 0.00 | 0.00 | 0.00 | 0.00 | 0.00 | 0.00 | 0.00 | 0.00 |
| alcohol_positive | 0.00 | 0.00 | 0.00 | 0.00 | 0.00 | 0.00 | 0.00 | 0.00 | 0.00 | 0.00 | 0.00 |
| any_od_lab_ordered_resulted | 0.01 | 0.01 | 0.01 | 0.01 | 0.01 | 0.01 | 0.01 | 0.01 | 0.01 | 0.00 | 0.01 |
| uds_amphetamine_methamphetamine_positive | 0.00 | 0.00 | 0.00 | 0.00 | 0.00 | 0.00 | 0.00 | 0.00 | 0.00 | 0.00 | 0.00 |
| uds_barbiturates_positive | 0.00 | 0.00 | 0.00 | 0.00 | 0.00 | 0.00 | 0.00 | 0.00 | 0.00 | 0.00 | 0.00 |
| uds_benzodiazepines_positive | 0.00 | 0.00 | 0.00 | 0.00 | 0.00 | 0.00 | 0.00 | 0.00 | 0.00 | 0.00 | 0.00 |
| uds_cannabinoids_positive | 0.00 | 0.00 | 0.00 | 0.00 | 0.00 | 0.00 | 0.00 | 0.00 | 0.00 | 0.00 | 0.00 |
| uds_cocaine_positive | 0.00 | 0.00 | 0.00 | 0.00 | 0.00 | 0.00 | 0.00 | 0.00 | 0.00 | 0.00 | 0.00 |
| uds_methadone_positive | 0.00 | 0.00 | 0.00 | 0.00 | 0.00 | 0.00 | 0.00 | 0.00 | 0.00 | 0.00 | 0.00 |
| uds_opiates_positive | 0.00 | 0.00 | 0.00 | 0.00 | 0.00 | 0.00 | 0.00 | 0.00 | 0.00 | 0.00 | 0.00 |
| uds_ethanol_positive | 0.00 | 0.00 | 0.00 | 0.00 | 0.00 | 0.00 | 0.00 | 0.00 | 0.00 | 0.00 | 0.00 |
| uds_oxycodone_positive | 0.00 | 0.00 | 0.00 | 0.00 | 0.00 | 0.00 | 0.00 | 0.00 | 0.00 | 0.00 | 0.00 |
| any_uds_ordered_resulted | 0.03 | 0.02 | 0.02 | 0.03 | 0.02 | 0.02 | 0.02 | 0.03 | 0.02 | 0.03 | 0.02 |
| encounter_year | 0.02 | 0.02 | 0.02 | 0.01 | 0.02 | 0.02 | 0.02 | 0.02 | 0.02 | 0.01 | 0.02 |
| provider_sex_male | 0.01 | 0.01 | 0.01 | 0.01 | 0.01 | 0.01 | 0.01 | 0.01 | 0.01 | 0.01 | 0.01 |
| Antidepressants | 0.01 | 0.01 | 0.01 | 0.01 | 0.01 | 0.01 | 0.01 | 0.01 | 0.01 | 0.01 | 0.01 |
| Antiepileptics | 0.00 | 0.00 | 0.00 | 0.00 | 0.00 | 0.00 | 0.00 | 0.00 | 0.00 | 0.00 | 0.00 |
| Antihistamines | 0.00 | 0.00 | 0.00 | 0.00 | 0.00 | 0.00 | 0.00 | 0.00 | 0.00 | 0.00 | 0.00 |
| Antipsychotics | 0.01 | 0.01 | 0.00 | 0.01 | 0.00 | 0.01 | 0.01 | 0.00 | 0.01 | 0.01 | 0.01 |
| Anxiolytics | 0.00 | 0.00 | 0.00 | 0.00 | 0.00 | 0.00 | 0.00 | 0.00 | 0.00 | 0.01 | 0.00 |
| Hypnotics | 0.00 | 0.00 | 0.00 | 0.00 | 0.00 | 0.00 | 0.00 | 0.00 | 0.00 | 0.00 | 0.00 |
| Lithium | 0.00 | 0.00 | 0.00 | 0.00 | 0.00 | 0.00 | 0.00 | 0.00 | 0.00 | 0.00 | 0.00 |
| Psychostimulants | 0.00 | 0.00 | 0.00 | 0.00 | 0.00 | 0.00 | 0.00 | 0.00 | 0.00 | 0.00 | 0.00 |
| Injectables | 0.00 | 0.00 | 0.00 | 0.00 | 0.00 | 0.00 | 0.00 | 0.00 | 0.00 | 0.00 | 0.00 |
| Number_of_med_classes | 0.01 | 0.01 | 0.01 | 0.01 | 0.01 | 0.01 | 0.01 | 0.01 | 0.01 | 0.01 | 0.01 |
